# Selective endocytosis of Ca^2+^-permeable AMPARs by the Alzheimer’s disease risk factor CALM bidirectionally controls synaptic plasticity

**DOI:** 10.1126/sciadv.abl5032

**Published:** 2022-05-25

**Authors:** Domenico Azarnia Tehran, Gaga Kochlamazashvili, Niccolò P. Pampaloni, Silvia Sposini, Jasmeet Kaur Shergill, Martin Lehmann, Natalya Pashkova, Claudia Schmidt, Delia Löwe, Hanna Napieczynska, Arnd Heuser, Andrew J. R. Plested, David Perrais, Robert C. Piper, Volker Haucke, Tanja Maritzen

**Affiliations:** 1 Leibniz-Forschungsinstitut für Molekulare Pharmakologie (FMP), Robert-Roessle-Straße 10, 13125 Berlin, Germany.; 2 Institute of Biology, Cellular Biophysics, Humboldt Universität zu Berlin, 10115 Berlin, Germany.; 3 University of Bordeaux, Interdisciplinary Institute for Neuroscience, UMR 5297, Bordeaux, France.; 4 CNRS, Interdisciplinary Institute for Neuroscience, UMR 5297, Bordeaux, France.; 5 Department of Nanophysiology, Technische Universität Kaiserslautern, Paul-Ehrlich-Strasse 23, 67663 Kaiserslautern, Germany.; 6 Department of Molecular Physiology and Biophysics, Carver College of Medicine, University of Iowa, Iowa City, IA 52242, USA.; 7 Animal Phenotyping, Max Delbrück Center for Molecular Medicine, Robert-Roessle-Straße 10, 13125 Berlin, Germany.; 8 NeuroCure Cluster of Excellence, Charité Universitätsmedizin Berlin, Virchowweg 6, 10117 Berlin, Germany.; 9 Freie Universität Berlin, Faculty of Biology, Chemistry and Pharmacy, 14195 Berlin, Germany.

## Abstract

AMPA-type glutamate receptors (AMPARs) mediate fast excitatory neurotransmission, and the plastic modulation of their surface levels determines synaptic strength. AMPARs of different subunit compositions fulfill distinct roles in synaptic long-term potentiation (LTP) and depression (LTD) to enable learning. Largely unknown endocytic mechanisms mediate the subunit-selective regulation of the surface levels of GluA1-homomeric Ca^2+^-permeable (CP) versus heteromeric Ca^2+^-impermeable (CI) AMPARs. Here, we report that the Alzheimer’s disease risk factor CALM controls the surface levels of CP-AMPARs and thereby reciprocally regulates LTP and LTD in vivo to modulate learning. We show that CALM selectively facilitates the endocytosis of ubiquitinated CP-AMPARs via a mechanism that depends on ubiquitin recognition by its ANTH domain but is independent of clathrin. Our data identify CALM and related ANTH domain–containing proteins as the core endocytic machinery that determines the surface levels of CP-AMPARs to bidirectionally control synaptic plasticity and modulate learning in the mammalian brain.

## Teaser

The Alzheimer’s disease risk factor CALM controls synaptic plasticity by a GluA1-specific endocytic mechanism.

## INTRODUCTION

Hebbian plasticity, which forms the cellular basis of experience-dependent learning and memory, involves bidirectional changes in synaptic strength via long-term potentiation (LTP) and long-term depression (LTD) (*1*). A primary mechanism in the plastic control of synaptic strength is an alteration in the number and composition of AMPA-type glutamate receptors (AMPARs), the main mediators of postsynaptic currents at excitatory synapses (*1*, *2*). The major form of LTP in the hippocampus originates from increased postsynaptic AMPAR levels within the postsynaptic density (PSD) that are supplied by nonsynaptic pools of diffusing AMPARs (*3*). These nonsynaptic AMPARs are replenished by exocytic delivery from recycling endosomes, which is balanced by endocytic AMPAR removal. Increased AMPAR delivery for LTP is controlled by posttranslational modifications such as phosphorylation of GluA1 (*4*) and by AMPAR-associated proteins that regulate AMPAR dynamics (*1*). Conversely, during LTD, synaptic AMPAR content is decreased by local depletion of surface AMPARs via endocytic mechanisms thought to depend on clathrin and its associated factors (*1*, *2*).

**Fig. 1.**
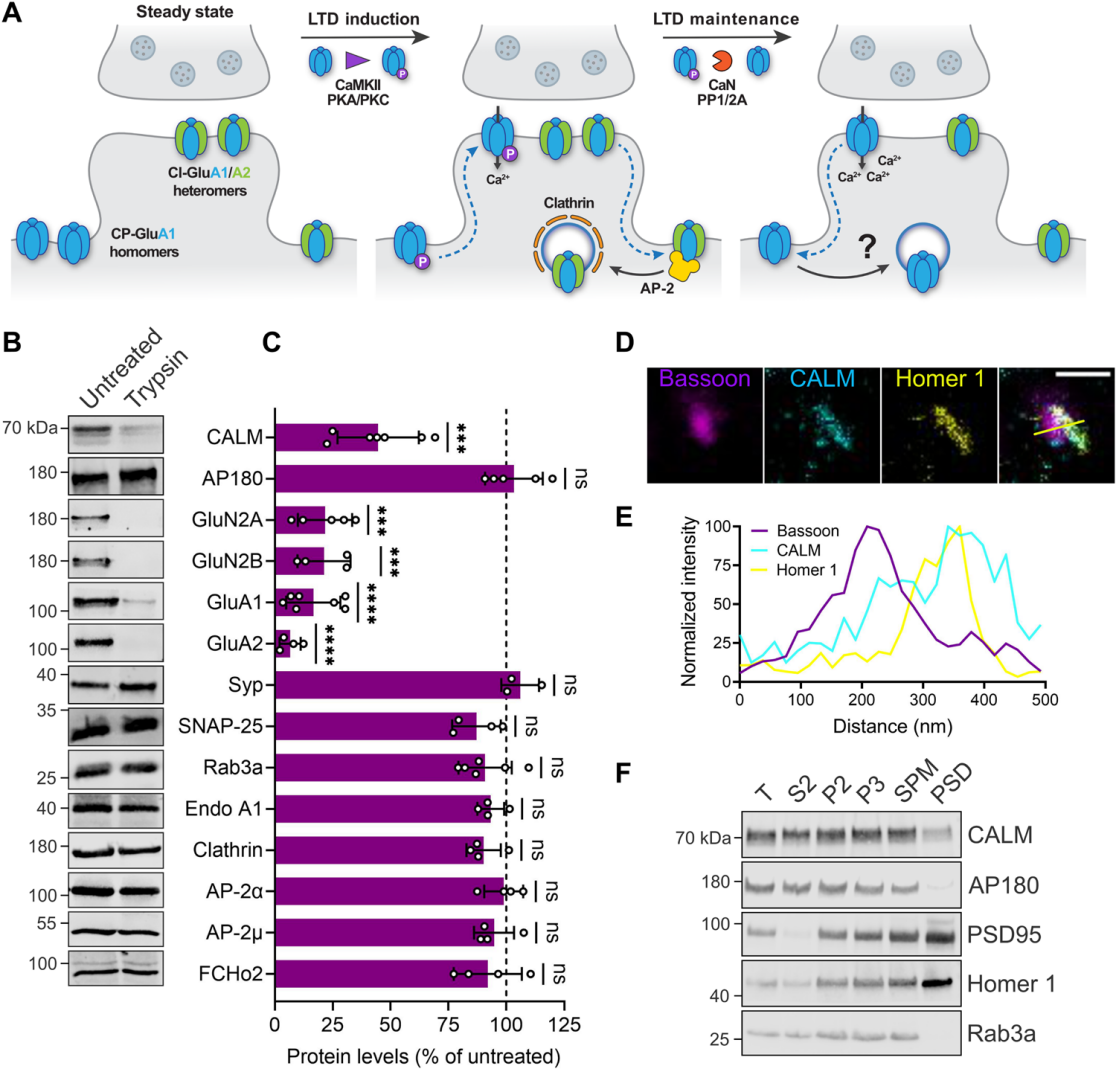
The clathrin adaptor CALM is enriched at postsynaptic endocytic zones where AMPAR endocytosis occurs. (**A**) During LTD induction, CP-AMPARs are recruited into synaptic regions, whereas CI-AMPARs are internalized via CME by binding to AP-2. Within a few minutes of LTD progression, the increased Ca^2+^ concentration activates phosphatases inducing GluA1 dephosphorylation and subsequent CP-AMPAR removal by a still unknown endocytic mechanism. (**B** and **C**) Tryptic digest of synaptosomes reveals postsynaptic localization of CALM. (B) Synaptosomes were left untreated or incubated with trypsin and analyzed by immunoblotting. (C) For quantification, trypsin-treated samples were normalized to untreated controls. (**D** and **E**) Three-channel time-gated STED confirms predominant postsynaptic localization of CALM. (D) Representative image of wild-type (WT) hippocampal neurons immunolabeled with antibodies against the presynaptic marker Bassoon, the postsynaptic marker Homer 1, and CALM (scale bar, 500 nm). Yellow line indicates position of the normalized fluorescent intensity depicted in (E). (**F**) The majority of CALM resides outside of the PSD. WT mouse brain homogenates were subjected to subcellular fractionation, and equal protein amounts of total homogenate (T), cytosolic fraction (S2), synaptosomes (P2), synaptosomal membranes (P3), synaptic plasma membranes (SPMs), and PSD were compared by immunoblotting with the indicated markers. Statistics Table 1. ns, not significant.

The functional diversity and computational power of excitatory synapses is further expanded by the fact that AMPARs are tetrameric assemblies of four different subunits termed GluA1 to GluA4, which confer distinct channel properties (*5*), thereby adding an additional yet poorly understood layer of regulation. The most common combinations of AMPARs at hippocampal CA1 synapses are GluA1/2 and GluA2/3 heteromers (*6*) that are impermeable to Ca^2+^ (CI-AMPARs), while a smaller population of GluA1 homomers is Ca^2+^-permeable (CP-AMPARs), thereby conveying distinctive signaling properties to the synapse. CP-AMPARs are broadly expressed during development, but their expression becomes more restricted in the adult brain (*7*) where they trigger spatiotemporally controlled Ca^2+^-dependent signaling events that prime synapses for both LTP (*8*, *9*) and LTD (*10*, *11*). During the induction phase of *N*-methyl-d-aspartate (NMDA) receptor (NMDAR)–dependent LTD, the initial Ca^2+^ transient triggers the phosphorylation-dependent recruitment of CP-AMPARs (i.e., GluA1 homomers) to the PSD (*11*), whereas CI-AMPARs (i.e., GluA2-containing heteromers) are removed by endocytosis (*4*, *12*). As LTD progresses, the sustained increase in Ca^2+^ activates the Ca^2+^-dependent phosphatase calcineurin to induce GluA1 dephosphorylation and CP-AMPAR removal (Fig. 1A). Failure to terminate GluA1 signaling during this phase impairs LTD (*11*), indicating that GluA1 subtype-selective internalization underlies NMDAR-dependent hippocampal LTD. Similar mechanisms may also form the basis of β-amyloid (Aβ)–induced removal of GluA1 and synaptic depression in Alzheimer’s disease (AD) (*13*–*15*). Despite its importance and in contrast to our detailed knowledge regarding the endocytosis of GluA2-containing CI-AMPARs, the mechanisms and molecular determinants that control the surface levels of GluA1-homomeric CP-AMPARs during long-term plastic changes of synaptic strength and in AD are largely unknown (Fig. 1A).

Here, we report that the endocytic adaptor clathrin assembly lymphoid myeloid leukemia protein (CALM) controls the surface levels of CP-AMPARs and, by reciprocally regulating LTP and LTD, modulates learning. The gene encoding CALM (denoted PICALM) has been identified as a susceptibility locus for AD by genome-wide association studies in human patients (*16*). Studies in different cell types have shown that CALM serves as an endogenous modulator of γ-secretase localization and activity (*17*), facilitates the endocytic sorting of SNARE proteins (*18*–*20*), and is required for transferrin internalization in erythroblasts (*21*). We demonstrate that, in neurons, CALM is enriched at the postsynapse to selectively target ubiquitinated CP-AMPARs for endocytosis via a mechanism that depends on direct ubiquitin recognition by its AP180 N-terminal homology (ANTH) domain but, unexpectedly, is independent of clathrin. Our data show that CALM and related ANTH domain–containing proteins act as a clathrin-independent endocytic platform that tunes the surface levels of CP-AMPARs to shape synaptic plasticity and learning in the mammalian brain and, thereby, may contribute to AD.

### RESULTS

#### The postsynaptically enriched endocytic adaptor CALM bidirectionally controls long-term synaptic potentiation and depression and, thereby, spatial learning

During LTD induction, Ca^2+^-impermeable GluA2-containing AMPARs (CI-AMPARs) are internalized via conventional clathrin-mediated endocytosis (CME) involving clathrin, the clathrin adaptor AP-2 (*22*–*24*), and its binding partner PICK1 (*25*). In contrast, extrasynaptic Ca^2+^-permeable GluA1 homomers (CP-AMPARs) are initially recruited to synapses to supply additional Ca^2+^ during the induction phase of LTD (*11*). These CP-AMPARs need to be removed eventually in an ill-defined pathway involving posttranslational modifications (*4*) and endocytosis via so far unknown mechanisms (Fig. 1A) (*1*). To identify the endocytic machinery underlying GluA1 internalization during long-term synaptic plasticity, we conducted a small-scale screen based on tryptic digestion of isolated mouse synaptosomes to identify endocytic proteins selectively enriched at the postsynapse. During synaptosome purification, the presynaptic terminal reseals into an enclosed compartment that protects presynaptic proteins from proteolysis, while postsynaptic proteins remain susceptible to tryptic digest (*26*). Consistently, we found presynaptic proteins such as synaptophysin, SNAP-25, or Rab3A to be protected from proteolysis (Fig. 1, B and C). Most endocytic proteins including endophilin A1, clathrin, the clathrin adaptor AP-2, and the BAR domain-containing protein FCHo2 displayed a similar behavior, consistent with their known presynaptic enrichment (*27*). In contrast, CALM, an endocytic adaptor widely expressed in the brain (fig. S1A) and associated with AD (*16*), was sensitive to digestion with trypsin, indicative of a large postsynaptic pool similar to the AMPAR subunits GluA1 and GluA2 and the NMDAR subunits GluN2A and GluN2B (Fig. 1, B and C). CALM’s close relative AP180 remained intact (Fig. 1, B and C), in agreement with its reported exclusive presynaptic localization (*28*). Isotropic multicolor time-gated stimulated emission depletion microscopy (time-gated STED) confirmed the colocalization of CALM with postsynaptic Homer 1, whereas CALM was much less prominent at presynapses identified by the active zone protein Bassoon (Fig. 1, D and E). Since in our STED images (fig. S1B) the PSD and perisynaptic regions could not be unequivocally distinguished, we turned to a biochemical approach to more reliably characterize the postsynaptic localization of CALM. In contrast to PSD-95 and Homer 1, little CALM was detected in the isolated PSD fraction (Fig. 1F) that harbors the active postsynaptic AMPAR pool. This observation is consistent with the notion that mobile AMPARs are internalized from specialized endocytic zones that surround the PSD (*3*), but not from the PSD itself. These data identify CALM as an endocytic protein selectively enriched at postsynaptic regions from where AMPAR endocytosis occurs.

**Fig. 2.**
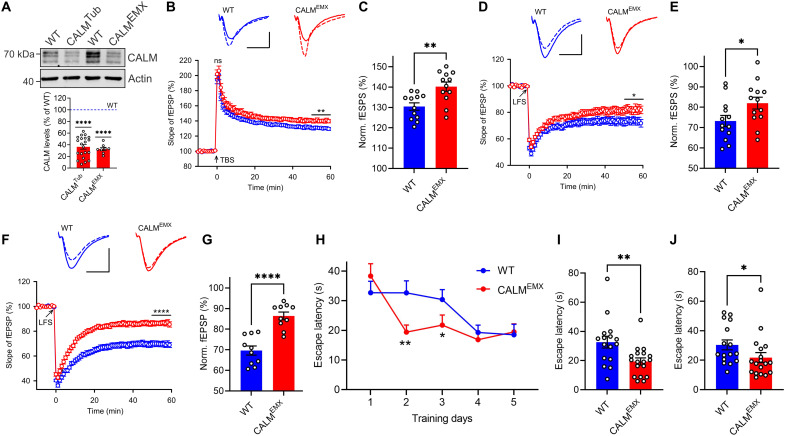
CALM bidirectionally controls synaptic plasticity and modulates learning. (**A**) Deletion of CALM in KO^Tub^ and KO^EMX^ mice. Brain lysates were analyzed by immunoblotting. Protein levels of CALM KO mice were normalized to WT littermates. (**B** and **C**) LTP induced by single theta-burst stimulation (TBS) is increased in 2-month-old CALM KO^EMX^ mice. (B) Graph shows normal presynaptic post-tetanic short-term potentiation measured immediately after TBS and increased postsynaptic LTP in CALM KO^EMX^ mice. Top: Representative fEPSPs recorded 0 to 10 min before (solid line) and 50 to 60 min after TBS (dashed line). Scale bar, 0.5 mV and 10 ms. (C) LTP values quantified as percent increase of the responses during the last 10 min. (**D** to **G**) LTD is reduced in 2-month-old CALM KO^EMX^ mice and severely impaired in 2-week-old CALM KO^EMX^ mice. (D and F) Top: Representative fEPSPs recorded 0 to 10 min before (solid line) and 50 to 60 min after LFS (dashed line). Scale bars, (D) 1 mV and 10 ms and (F) 0.5 mV and 10 ms. (E and G) LTD values quantified as percent decrease of the responses during the last 10 min. (**H** to **J**) CALM KO^EMX^ mice show improved spatial learning. (H) Average escape latency over 5 days of training. Depiction of individual escape latencies for days 2 (I) and 3 (J), where CALM KO^EMX^ mice outperformed controls. Statistics Table 1.

We hypothesized that CALM may constitute an essential element of the endocytic machinery that removes AMPARs during long-term plastic changes of neurotransmission, possibly in a subunit-selective fashion (see below). To test this hypothesis, we generated conditional knockout (KO) mice selectively lacking CALM expression in all neurons of the brain [CALM^lox/lox^ × Tubulin α1-Cre mice (*29*); denoted CALM^Tub^; fig. S1C] or only in postmitotic excitatory neurons of the cortex and hippocampus [CALM^lox/lox^ × EMX1-Cre (*30*); denoted CALM^EMX^; fig. S1D]. Both CALM KO mouse lines were born at the expected Mendelian ratio (fig. S1, C and D), and they were indistinguishable with respect to the efficacy of CALM deletion, as observed in brain lysates (~75% decrease in CALM levels; the remaining ~25% of CALM protein originates from non-neuronal cells in the central nervous system) (Fig. 2A). CALM KO mice from either line were also indistinguishable from their Cre-negative floxed control littermates [denoted as wild type (WT) in figures for brevity] with respect to overall brain anatomy (fig. S1E), postnatal weight development (fig. S1F), and the expression of other pre- and postsynaptic proteins (fig. S1, G and H). As an exception, we noted a small increase in the total levels of GluA2, possibly reflecting a compensatory mechanism to reduce excitotoxicity in vivo (see below).

We first analyzed whether loss of CALM affects basal synaptic transmission by recording field excitatory postsynaptic potentials (fEPSPs) of Schaffer collateral CA1 synapses in acute hippocampal slices from 2- to 3-month-old mice. Analyses of input-output curves by plotting the slope of fEPSP versus fiber volley amplitudes in response to a range of stimulus intensities indicated no detectable change in basal synaptic transmission (fig. S2A). Consistent with the prominent postsynaptic localization of CALM, we found no alteration in paired-pulse facilitation (PPF) in CALM KO^EMX^ mice (fig. S2B), a surrogate measure of presynaptic release probability, or in post-tetanic short-term potentiation (Fig. 2B), which occurs immediately after LTP conditioning and lasts for ~2 min. Moreover, neuron-specific loss of CALM did not lead to changes in anxiety-related behavior in the elevated plus maze (fig. S2, C to E). Thus, neuronal loss of CALM does not recapitulate any of the prominent presynaptic phenotypes observed in KO mice lacking its close relative AP180 (*28*), which is presynaptically enriched. Overall, these data indicate that neuronal CALM is largely dispensable for brain development and basal neurotransmission.

To probe for a possible role of CALM in the endocytic removal of AMPARs during long-term changes of neurotransmission, we analyzed postsynaptic LTP and LTD. LTP following single theta-burst stimulation (TBS) was significantly increased at synapses from CALM KO^EMX^ mice (Fig. 2, B and C). Conversely, we found NMDAR-dependent LTD induced by low-frequency stimulation (LFS) to be reduced in 2-month-old CALM KO^EMX^ mice compared to controls (Fig. 2, D and E). This phenotype was more pronounced in slices from 2-week-old CALM KO^EMX^ mice (Fig. 2, F and G; see also fig. S2F for basal transmission), in line with the fact that juvenile mice are known to display comparably higher GluA1 levels and stronger LTD (*31*, *32*). In contrast, in AP180 KO mice, depression of synaptic responses was unaltered (fig. S2G). Hence, CALM antagonistically controls postsynaptic LTP and LTD at hippocampal synapses.

To probe whether the observed changes in postsynaptic plasticity affect hippocampus-dependent learning of CALM KO mice, we monitored spatial learning using the Morris water maze. During visible platform trials, control and CALM KO^EMX^ mice readily learned to find the marked platform. Mice were then trained to swim to a hidden platform located in a fixed location of the pool. While there was no difference between genotypes in swimming speed (fig. S2H) and during the probe trial on day 5 (fig. S2I), CALM KO^EMX^ mice showed an improvement in spatial learning (Fig. 2H and fig. S2J) on days 2 and 3 of the training, as evidenced by a shorter escape latency (Fig. 2, I and J). Together, these findings indicate that the postsynaptically enriched endocytic protein CALM antagonistically controls long-term synaptic potentiation (see model in fig. S2K) and depression and, thereby, modulates spatial learning in a hippocampus-dependent paradigm.

#### Elevated levels of synaptic CP-AMPARs underlie elevated LTP and reduced LTD in the absence of CALM

Changes in the postsynaptic abundance of AMPARs are a major mechanism for controlling LTP and LTD (*1*, *3*). GluA1 KO mice lack LTP in the hippocampal CA1 region (*33*), and partitioning of GluA1-containing AMPARs between intracellular, extrasynaptic, and synaptic pools plays a critical role in LTP and LTD (*3*, *11*) and modulates learning (*34*).

**Fig. 3.**
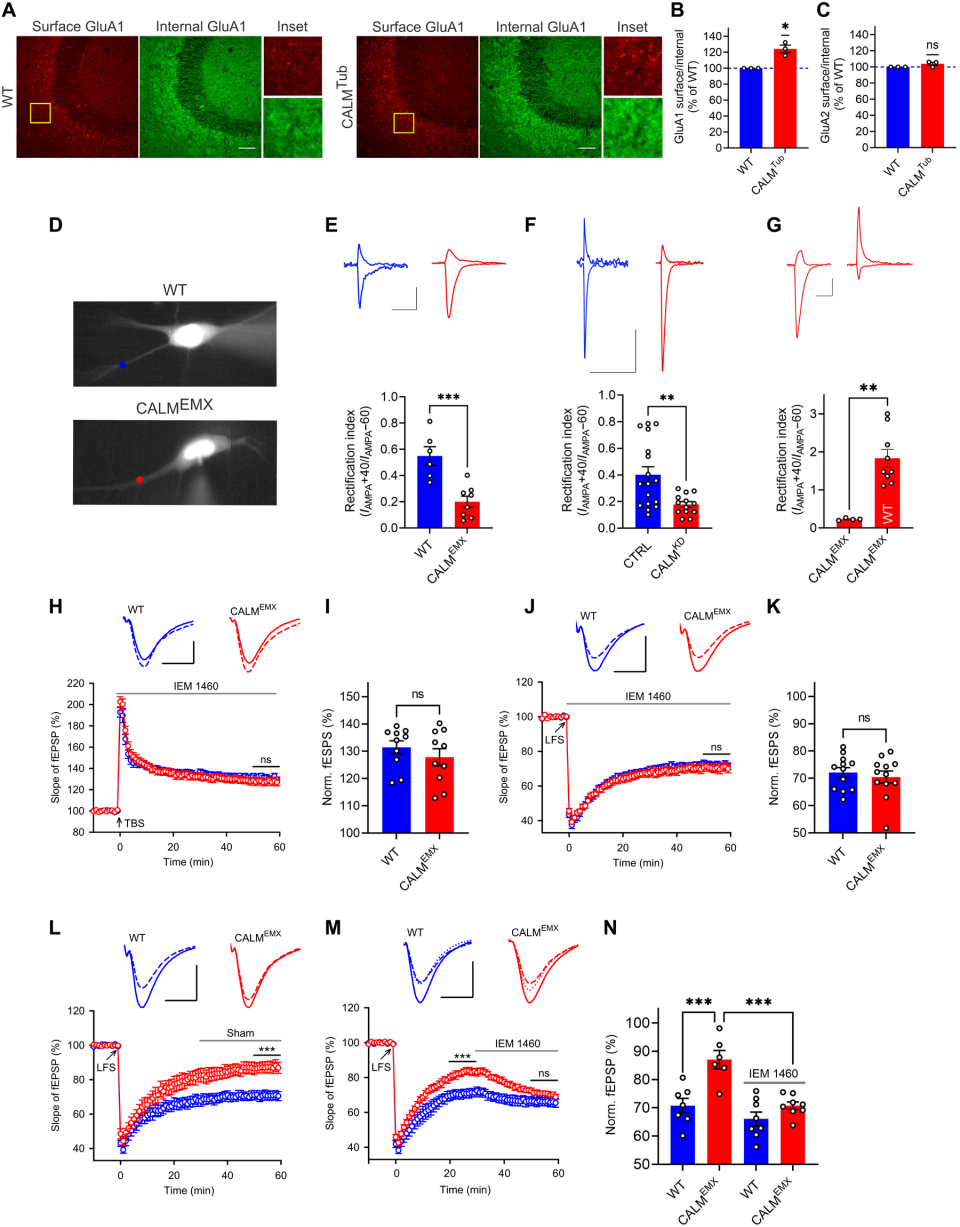
Surface accumulation of CP-AMPARs in CALM KO mice underlies synaptic plasticity deficits. (**A** to **C**) CALM KO elevates GluA1 surface pools. (A) Brain sections immunostained for GluA1 in the absence/presence of detergent to label surface or internal pool (scale bar, 50 μm). Surface/total ratios of GluA1 (B) or GluA2 (C) normalized to WT. (**D**) Neurons where AMPAR surface levels at synapses were monitored after glutamate uncaging. (**E** and **F**) CALM KO (E) or knockdown (F) leads to decreased AMPAR rectification index (scale bars, 30 pA and 300 ms). (**G**) Reexpression of CALM in KO^EMX^ slices not only rescues increased inward rectification but also causes outward rectification (scale bars, 30 pA and 300 ms). (**H** to **K**) Increased LTP (H) and impaired LTD (J) in CALM-deficient mice are rescued by IEM 1460, applied after induction. Top: fEPSPs recorded 0 to 10 min before (solid) and 50 to 60 min after TBS or LFS (dashed). Scale bars, 0.5 mV and 10 ms (TBS) and 1 mV and 10 ms (LFS). LTP (I) or LTD (K) values quantified as percent change of the responses (last 10 min). (**L** to **N**) Impaired LTD in CALM-deficient mice (L) is rescued by IEM 1460, applied 30 min after LTD induction (M). Top: Representative fEPSPs recorded 0 to 10 min before LFS (solid), 20 to 30 min after LFS (before IEM 1460 application; dotted), and 50 to 60 min after LFS (dashed) (scale bars, 1 mV and 10 ms). (N) LTD values quantified as percent change of the responses (last 10 min). Statistics Table 1.

We therefore hypothesized that the observed elevation in LTP and reduction in LTD in CALM KO mice might result from altered surface levels of GluA1- and/or GluA2-containing AMPARs in vivo. To specifically detect the surface levels of GluA1 and GluA2, we performed immunohistochemistry on brain sections from CALM KO^Tub^ and control mice under nonpermeabilizing conditions using antibodies that specifically recognize the extracellular domain of GluA1 or GluA2, and in the presence of detergent, to assess GluA1 and GluA2 total levels. This analysis revealed a small but significant elevation of the surface-to-total GluA1 ratio (Fig. 3, A and B), while GluA2 surface levels were unaffected (Fig. 3C and fig. S3A). These data suggest that loss of neuronal CALM causes the partial repartitioning of GluA1-containing AMPARs to the neuronal surface. Conceivably, these may increase the synaptic pools of GluA1-homomeric CP-AMPARs and/or that of GluA1/2-heteromeric CI-AMPARs (*6*, *35*). To distinguish between these possibilities, we conducted patch clamp recordings of organotypic slice cultures, in which AMPAR surface levels can be monitored upon glutamate uncaging at dendrites (Fig. 3D) (*36*). As surface-localized GluA1-homomeric CP-AMPARs are sensitive to a block of their outward currents at positive holding potentials by intracellular polyamines leading to pronounced inward rectification (*37*), they can be readily distinguished from GluA2-containing CI-AMPARs. Measurements of the rectification index (*I*_AMPA_+40/*I*_AMPA_−60) as a readout of the levels of CP-AMPARs in organotypic slices from control versus CALM KO^EMX^ mice revealed a markedly reduced rectification index in the absence of CALM upon glutamate uncaging (Fig. 3E). A similar reduction of the rectification index was observed in organotypic slices from WT mice, in which CALM had been depleted by lentiviral short hairpin RNA (shRNA; Fig. 3F and fig. S3B) and in acute slices from CALM KO^EMX^ mice in response to electrical stimulation of endogenous synaptic glutamate release at Schaffer collateral CA1 synapses (fig. S3C). Together, these experiments are indicative of increased CP-AMPAR surface levels in dendrites of CALM KO^EMX^ mice. Overexpression of CALM by adeno-associated virus (AAV) 2/9–based transduction of CALM KO^EMX^ organotypic slices not only rescued decreased rectification but also resulted in a pronounced outward rectification (Fig. 3G), consistent with a depletion of CP-AMPARs. These data provide evidence that CALM controls the surface levels of functional GluA1 homomers and suggest that the resulting excess of synaptic CP-AMPARs underlies elevated LTP and defective LTD expression in CALM KO^EMX^ mice. Consistent with this model, we found that specific inhibition of GluA1 homomers by IEM 1460, a selective voltage-dependent open-channel CP-AMPAR inhibitor, applied after induction (*11*), reverted LTP (Fig. 3, H and I) and LTD expression (Fig. 3, J and K) in slices from CALM KO^EMX^ mice to that of WT. Applying IEM 1460 only during the late phase of LTD (i.e., 30 min after induction) also restored LTD to normal WT levels in CALM KO^EMX^ slices (Fig. 3, L to N). In contrast, IEM 1460 did not have a significant effect on basal synaptic transmission in WT or CALM KO^EMX^ slices (fig. S3, D to F). These data support the notion that GluA1 homomers shape synaptic plasticity but are largely dispensable for basal synaptic transmission. In summary, our results show that CALM controls the surface and, thereby, the synaptic levels of CP-AMPARs to antagonistically regulate synaptic long-term plasticity in vivo.

#### Loss of neuronal CALM selectively impairs the endocytosis of GluA1 homomers in hippocampal neurons

**Fig. 4.**
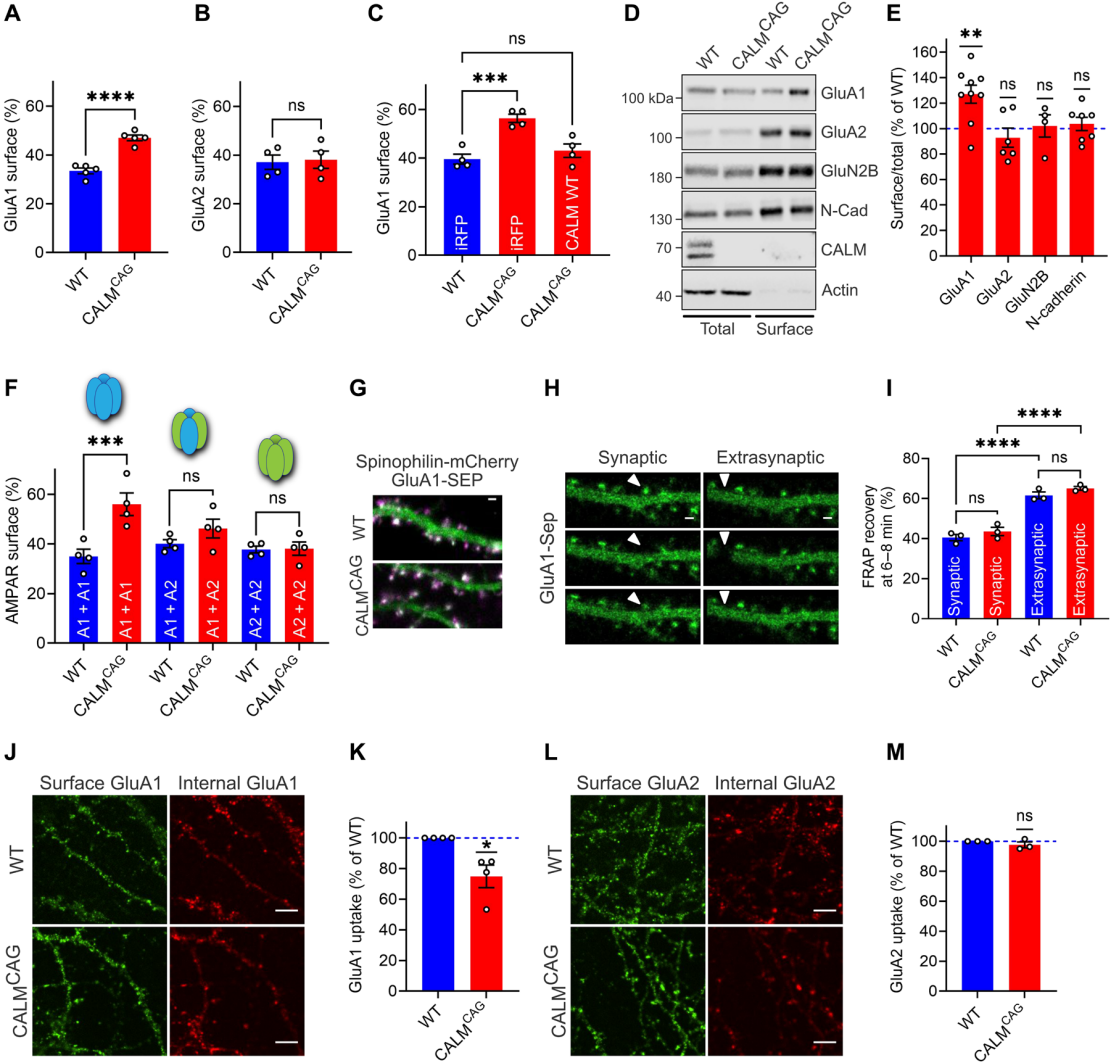
Loss of neuronal CALM selectively impairs the endocytosis of GluA1 homomers. (**A** and **B**) GluA1 is missorted upon CALM KO. Surface/total ratios of SEP-GluA1 (A) or SEP-GluA2 (B) expressed in WT or KO^CAG^ neurons. (**C**) Rescue of increased GluA1 surface level in KO^CAG^ neurons by CALM reexpression. Surface/total protein ratios of SEP-GluA1 in neurons transduced with pAAV-iRFP or pAAV-CALM-WT. (**D** and **E**) CALM deletion causes increased surface levels of endogenous GluA1. After surface biotinylation of neurons, total and biotinylated proteins were analyzed by immunoblotting (D). Surface/total ratios normalized to WT (E). (**F**) CALM KO causes the accumulation of GluA1 homomers. Surface/total protein ratios of SEP-tagged GluA1 or GluA2 in WT or KO^CAG^ neurons cotransfected with HA-GluA1 or HA-GluA2 to unravel differences between homomers and heteromers. (**G** to **I**) Surface accumulation of GluA1 upon CALM loss not due to altered exocytosis or lateral diffusion. WT and CALM KO^CAG^ neurons coexpressing SEP-GluA1 (green) and dendritic mCherry-spinophilin (magenta) (G) were photobleached at synaptic and extrasynaptic regions (H) (scale bar, 1 μm). Percent fluorescence recovery at 6 to 8 min after beaching (I). (**J** to **M**) GluA1 endocytosis is impaired in the absence of CALM. WT and CALM KO^CAG^ neurons live-labeled using antibodies recognizing GluA1 (J) or GluA2 (L) were fixed and immunostained in the absence/presence of detergent to label the surface or internal pool (images: scale bar, 5 μm). GluA1 (K) or GluA2 (M) uptake normalized to WT. Statistics Table 1.

What is the molecular mechanism by which CALM controls GluA1 surface partitioning to regulate synaptic plasticity? We addressed this important question with the help of a third animal model generated by crossing CALM^lox/lox^ mice with a tamoxifen-inducible Cre line (fig. S4A) (*38*). To probe the effects of acute tamoxifen-induced loss of CALM (fig. S4B) on AMPAR surface pools, we expressed GluA1 or GluA2 tagged with a pH-sensitive green fluorescent protein (GFP) variant [superecliptic pHluorin (SEP)] in control and CALM KO^CAG^ hippocampal neurons and monitored their partitioning between internal acidic compartments (e.g., endosomes) and the cell surface using an established acid quenching/dequenching protocol (fig. S4C). Consistent with our findings in acute slices, CALM KO^CAG^ hippocampal neurons exhibited a selective elevation in the fraction of nonretrieved GluA1-containing AMPARs (Fig. 4A), while the surface levels of GluA2 remained unaltered (Fig. 4B). A similar phenotype was observed when CALM was depleted from WT neurons using shRNA delivered by lentivirus (fig. S4, B, D, and E). GluA1 surface accumulation was perfectly rescued by AAV2/9-mediated reexpression of full-length CALM protein (Fig. 4C and fig. S4F). In line with its presynaptic localization, loss of the CALM-related adaptor AP180 did not affect GluA1 surface levels (fig. S4, G to I). In agreement with the fact that CALM loss does not alter presynaptic release probability in acute slices (fig. S2B), CALM deficiency had no effect on the endocytic sorting or surface partitioning of the presynaptic vesicle protein synaptophysin (fig. S4, J and K). These data suggest that postsynaptic CALM specifically controls the surface levels of GluA1-containing AMPARs. To provide direct evidence for the regulation of native receptors by CALM, we monitored the plasma membrane levels of endogenous GluA1 and GluA2 by biotinylation. Tagging the plasma membrane proteome of cultured cortico-hippocampal neurons with sulfo-NHS-SS-biotin confirmed the selective surface enrichment of GluA1, but not GluA2, in CALM KO^CAG^ (Fig. 4, D and E) or CALM^KD^ neurons (fig. S4L) without any apparent alteration in the total protein levels of GluA1 (fig. S4, M and N). Neither the surface nor the total protein levels of other plasma membrane proteins including GluN2B, an NMDAR subunit, or N-cadherin were affected by loss of CALM (Fig. 4, D and E, and fig. S4, L to N). In agreement, we found no alteration in NMDAR function in CALM KO^EMX^ mice when calculating NMDA/AMPA ratios (fig. S4, O and P).

Although these data demonstrate that postsynaptic CALM specifically controls the surface levels of GluA1-containing AMPARs, they do not allow us to unequivocally distinguish between surface accumulation of GluA1 homomers and GluA1/2 heteromers (*6*, *39*). To address this point, we compared neurons expressing GluA1 or GluA2 either alone [which form homomers, as confirmed by (*40*)] or in combination to foster GluA1/A2 heteromer formation. Conditional KO of CALM led to a pronounced surface accumulation of overexpressed GluA1 homomers and caused a small yet nonsignificant increase (*P* = 0.4922) in the surface abundance of GluA1/2-heteromeric AMPARs induced by the combined expression of GluA1 and GluA2 (Fig. 4F). In contrast, the surface pool of GluA2 homomers was unaffected by CALM loss. Heteromeric GluA1/2 and homomeric GluA2 AMPARs likely rely predominantly on the association of the GluA2 subunit with the clathrin-based endocytic machinery via AP-2 (Fig. 1A) (*22*, *23*) and may thus be less affected by loss of CALM. Together with our electrophysiological recordings in acute and organotypic slices, these results show that CALM controls the surface levels of GluA1 by preferentially recognizing a comparably small population of CP-AMPARs, i.e., GluA1 homomers, that play a pivotal role in synaptic plasticity.

At least two different mechanisms can be envisaged by which CALM controls GluA1 levels on the neuronal surface: Conceivably, (i) CALM might regulate GluA1 mobility, a key process that controls AMPAR function during plasticity (*3*, *34*). This could occur, for example, by affecting GluA1 recruitment and anchoring to the PSD either directly or by altering the posttranslational modification of GluA1. Dephosphorylation of GluA1-S845, for example, is required for GluA1 removal from the postsynaptic membrane during LTD (*41*). Alternatively, (ii) CALM might serve a neuron-specific function in the selective recognition of GluA1 to facilitate its endocytic internalization from the postsynaptic membrane.

To address a possible role for CALM in the exocytic insertion, recruitment, or diffusion of GluA1, we performed FRAP (fluorescence recovery after photobleaching) experiments to determine the mobility of fluorescently tagged GluA1 in spines and dendritic shafts (Fig. 4, G and H, and fig. S5A). While GluA1 was found to be more mobile in extrasynaptic areas compared to synapses, FRAP recovery of GluA1 was unaffected by CALM loss (Fig. 4I and fig. S5B). Likewise, we failed to detect any difference in the levels of GluA1-pS845 upon LTD induction between control and CALM KO neurons (fig. S5, C and D). These data argue against a causative role for CALM in regulating GluA1 insertion, mobility, or de/phosphorylation at S845. We therefore followed the alternative possibility that CALM selectively facilitates the endocytosis of GluA1 from the postsynaptic membrane. To this end, we monitored the endocytosis of endogenous GluA1- or GluA2-containing AMPARs from the neuronal surface using an antibody internalization assay. Consistent with our data from pHluorin-based imaging experiments, the internalization of endogenous GluA1 (Fig. 4, J and K), but not of GluA2 (Fig. 4, L and M), was reduced in CALM KO^CAG^ neurons. The comparably small effect size in this assay [compare also Fig. 3 (A and B) and Fig. 4 (D and E)] likely is a reflection of the selective role of CALM in controlling the surface levels of CP-AMPARs consisting of GluA1 homomers, which constitute a minor fraction of the total GluA1-containing AMPAR pool (*6*, *39*). We also probed the effects of NMDA stimulation on AMPAR endocytosis in WT and CALM KO^CAG^ neurons. NMDA application had no major effect on the efficacy of GluA1 endocytosis (fig. S5, E and F), while it increased the intracellular accumulation of GluA2 (fig. S5, G and H), consistent with earlier data (*42*). GluA1 internalization remained CALM dependent in the presence of NMDA (fig. S5, E and F). In contrast to the inhibition of GluA1 homomer internalization, CALM loss did not affect the endocytosis of the general CME cargo transferrin (fig. S5, I to L) or the partitioning of transferrin receptors tagged with pHuji, a pH-sensitive red fluorescent protein (RFP), between acidic endosomal compartments and the cell surface (fig. S5, M and N). Collectively, our findings unravel a neuron-specific role for CALM in the bidirectional regulation of long-term synaptic plasticity by selectively facilitating the endocytosis of CP-AMPARs (i.e., GluA1 homomers) and, possibly to some extent, GluA1-containing heteromers and their associated factors.

#### GluA1 endocytosis is driven by CALM-mediated membrane remodeling and is independent of clathrin

**Fig. 5.**
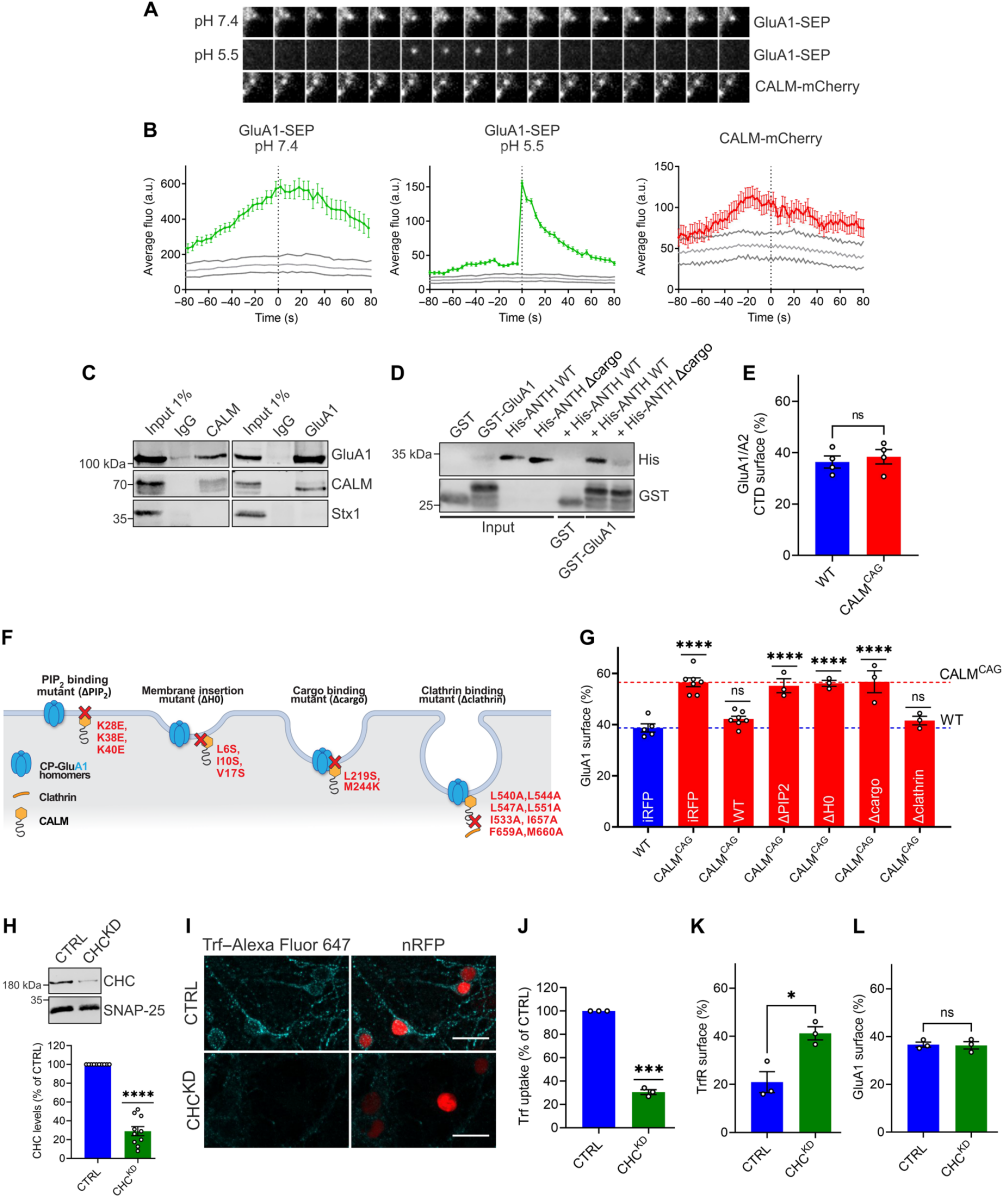
GluA1 endocytosis is driven by CALM-mediated membrane remodeling and independent of clathrin. (**A** and **B**) CALM-mCherry is present during GluA1 endocytosis events detected with ppH assay at pH 5.5 (A). (B) Average fluorescence intensity aligned to vesicle detection time (frame 0; gray areas, 95% confidence interval of randomized data). (**C**) Coimmunoprecipitation of endogenous GluA1-CALM complexes from synaptosomal membranes using GluA1- or CALM-specific antibodies. (**D**) CALM-ANTH domain binds cytosolic GluA1–C terminus. GST–GluA1–C terminus or GST was incubated with His-tagged WT-CALM-ANTH or cargo binding–deficient mutant (Δcargo). Analysis by immunoblotting. (**E**) Surface levels of GluA1-chimera containing C-terminal domain of GluA2 are unaffected by CALM loss. Neuronal surface/total protein ratios of SEP-GluA1-chimera assessed by acid-base quenching. (**F**) CALM mutants of (G). (**G**) CALM’s function in GluA1 endocytosis depends on its PI(4,5)P_2_ binding and membrane curvature induction but is clathrin independent. SEP-GluA1 surface/total levels in neurons coexpressing iRFP (control), WT-CALM, or CALM-mutant deficient in PI(4,5)P_2_ binding, membrane insertion (ΔH0), GluA1 binding (Δcargo), or clathrin binding. (**H**) shRNA-mediated clathrin depletion. Lysates of WT neurons expressing scrambled or anti-CHC shRNA were probed by immunoblotting (clathrin protein levels normalized to SNAP-25). (**I** and **J**) Endocytosis of Alexa Fluor 647–transferrin is impaired in clathrin-depleted cells (images: scale bar, 20 μm; nuclear RFP, transduced cells). Values normalized to control cells. (**K**) Increased transferrin receptor surface pool in clathrin-depleted neurons expressing pHuji-TrfR. (**L**) Unaltered GluA1 surface pool in clathrin-depleted neurons expressing SEP-GluA1. Statistics Table 1. a.u., arbitrary units.

To explore the molecular mechanism and machinery that underlie CALM-mediated endocytosis of GluA1 homomers, we monitored the spatiotemporal dynamics of CALM during the endocytosis of SEP-GluA1 and SEP-GluA2 in a pulsed-pH (ppH) assay that capitalizes on a rapid exchange between low (5.5) and high pH (7.4) buffers, enabling the detection of AMPAR endocytic events. We observed CALM to colocalize with high spatial and temporal precision (i.e., ≤2 s) with GluA1-containing endocytic vesicles formed within postsynaptic dendrites and near synapses (Fig. 5, A and B, and fig. S6, A and B). CALM was also present at sites of GluA2 endocytosis (fig. S6, A, C, and D), despite the fact that CALM is dispensable for GluA2 internalization. These data support a model whereby cargo-selective endocytic adaptors operate at defined endocytic sites to facilitate the internalization of specific cargos (i.e., GluA1 in the case of CALM) but may be dispensable for the endocytic process per se.

These combined observations suggest that CALM may recruit CP-AMPARs to endocytic sites, likely by directly or indirectly associating with GluA1. To probe this, we performed immunoprecipitations of endogenous CALM from detergent-extracted synaptosomal membrane fractions. GluA1 coprecipitated with CALM (Fig. 5C). Conversely, antibodies against the extracellular domain of GluA1 coimmunoprecipitated CALM (Fig. 5C). These results demonstrate that endogenous GluA1 and CALM undergo complex formation in the brain in vivo.

Previous work has shown that CALM associates with charged plasma membrane lipids such as phosphatidylinositol 4,5-bisphosphate [PI(4,5)P_2_] (*43*) and with endocytic cargo proteins including vesicle-associated membrane protein (VAMP) family members via distinct binding sites in its so-called ANTH domain (*18*–*20*). We therefore hypothesized that neuronal CALM may directly associate with GluA1 via its ANTH domain. To test this, we incubated the recombinantly expressed glutathione *S*-transferase (GST)–tagged cytoplasmic tail of GluA1 with the purified His_6_-tagged ANTH domain of CALM (fig. S6E). We found CALM-ANTH to directly bind with moderate efficacy to GST-GluA1, but not to GST (Fig. 5D). Complex formation was abrogated if the known binding site for cargo proteins within the ANTH domain (*18*, *20*) was mutated (CALM-ANTH^Δcargo^) (Fig. 5D and fig. S6E). These results indicate that the ANTH domain of CALM is both required and sufficient to directly associate with the cytoplasmic tail of GluA1. In agreement with the hypothesis that the variable C-terminal domain of AMPARs is the site conferring distinct modes of regulation between the different subunits, a SEP-GluA1 chimera containing the C-terminal tail of GluA2 (GluA1/A2 CTD) failed to accumulate at the cell surface of CALM KO^CAG^ neurons (Fig. 5E), as revealed by acid quenching/dequenching experiments. These data show that CALM controls GluA1 surface levels by direct association of its ANTH domain with the cytoplasmic tail of GluA1.

Given the central role of the ANTH domain of CALM in GluA1 endocytosis, we aimed to further explore the molecular determinants that underlie its function. To this aim, we created mutant versions of CALM that selectively lack the ability to associate with PI(4,5)P_2_ (ΔPIP_2_) (*43*), to deform the plasma membrane via insertion of an amphipathic helix (ΔH0) (*19*), or to bind cargo proteins such as VAMPs (Δcargo) (Fig. 5F) (*18*, *20*). We then capitalized on a molecular replacement strategy based on AAV2/9-mediated reexpression of WT or mutant CALM variants to assess their ability to functionally rescue GluA1 surface accumulation in CALM KO^CAG^ neurons (fig. S6, F to H). As expected, reexpression of WT CALM restored GluA1 surface levels to those observed in hippocampal neurons from control mice (Fig. 5G; see also Fig. 4C). In contrast, mutant versions of CALM lacking the ability to bind to PI(4,5)P_2_ or to facilitate endocytic vesicle formation by membrane deformation or to associate with cargo proteins (i.e., GluA1) were incapable of reducing the GluA1 surface pool to WT levels (Fig. 5G). These data indicate that CALM controls GluA1 surface levels by spatiotemporally coupling local postsynaptic plasma membrane deformation with the direct recognition of GluA1 to mediate its sorting to nascent endocytic vesicles.

Recent work in *Caenorhabditis elegans* and in hippocampal neurons has shown that endocytic membrane internalization within the presynaptic compartment capitalizes on endocytic proteins such as endophilin and dynamin previously implicated in CME but is functionally independent of clathrin (*44*–*46*). We therefore explored whether the subunit-selective endocytic control of GluA1 surface levels by CALM requires clathrin. In vitro studies together with work in non-neuronal cells demonstrated that CALM potently binds to clathrin via a series of degenerate motifs. We used a CALM mutant deficient in clathrin binding (Δclathrin) (Fig. 5F) (*47*) to explore whether the ability to directly associate with clathrin is required to functionally rescue GluA1 surface accumulation in CALM KO^CAG^ neurons. Very much to our surprise, CALM^Δclathrin^ was perfectly capable of restoring defective GluA1 retrieval (Fig. 5G and fig. S6, F to H). Given this unexpected finding, we decided to challenge these results by an independent approach. We depleted hippocampal neurons of endogenous clathrin using an established lentiviral shRNA approach (*44*, *45*) that resulted in a severe reduction of clathrin levels to ~25 to 30% of those in controls (Fig. 5H). As expected, clathrin knockdown strongly impaired CME, as evidenced by a near complete loss of the ability of clathrin KD (CHC^KD^) hippocampal neurons to internalize fluorescently labeled transferrin, a bona fide CME cargo (Fig. 5, I and J). Moreover, clathrin KD (CHC^KD^) neurons suffered from a roughly twofold increase in the cell surface levels of transferrin receptor (Fig. 5K). In contrast to its effects on transferrin uptake, clathrin depletion did not alter the neuronal surface levels of GluA1 (Fig. 5L). These data, together with the fact that CALM loss selectively perturbs GluA1 endocytosis and surface partitioning, demonstrate that GluA1 endocytosis is driven by CALM-mediated membrane remodeling, yet is independent of clathrin, akin to presynaptic endocytosis of synaptic vesicle proteins (*44*, *45*).

#### CALM and related ANTH domain–containing proteins endocytose ubiquitinated GluA1 homomers by direct recognition of ubiquitin

**Fig. 6.**
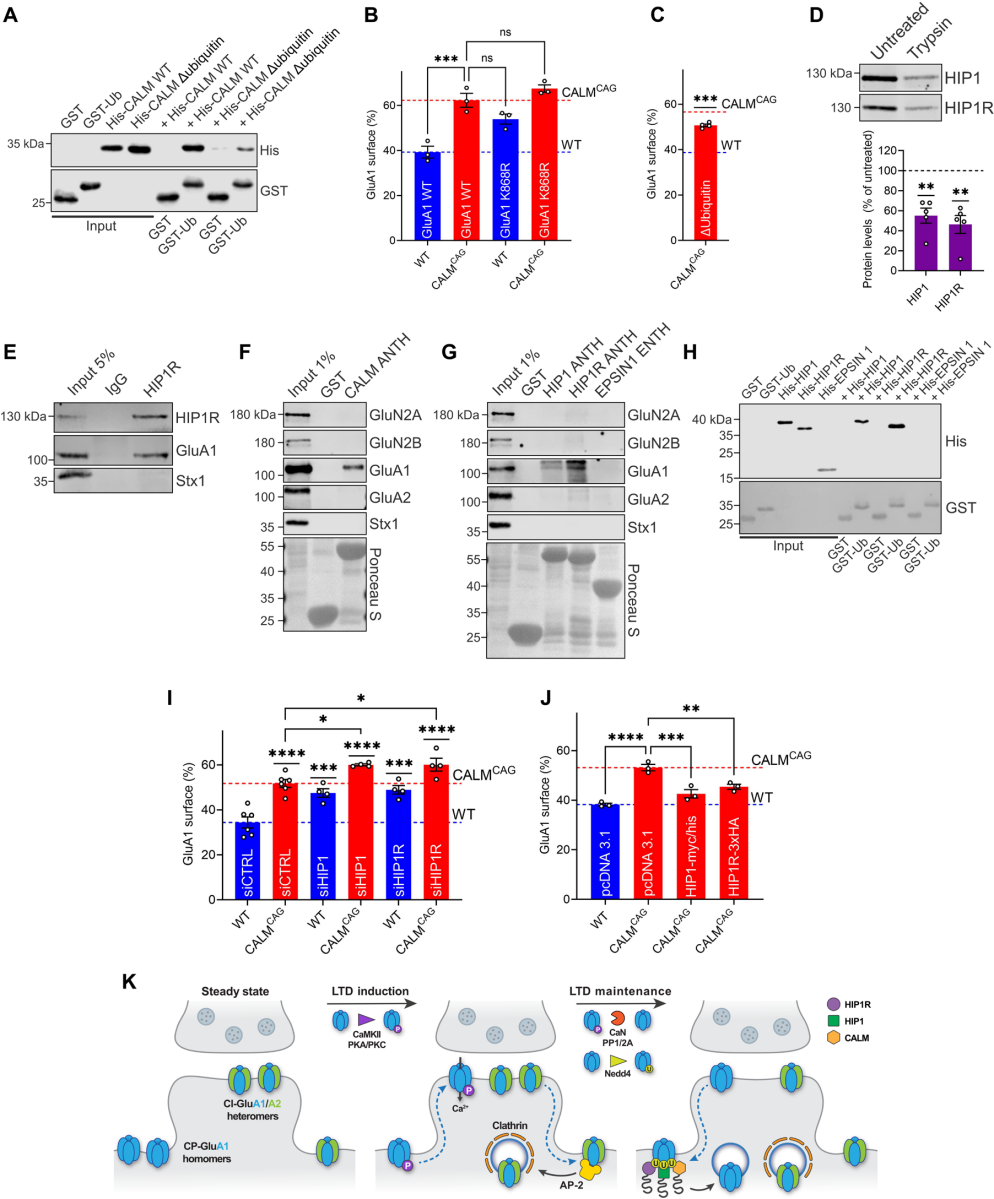
Sorting of GluA1 homomers is a general feature of ANTH domain–containing proteins and involves ubiquitin. (**A**) CALM-ANTH binds ubiquitin. GST-ubiquitin or GST was incubated with His-WT-CALM-ANTH or its ubiquitin binding–deficient mutant. Analysis by immunoblotting. (**B**) Ubiquitination and CALM-dependent GluA1 sorting act in the same pathway. Neuronal surface/total protein ratios of SEP-GluA1-WT or its ubiquitination-deficient K868R mutant. (**C**) Ubiquitin binding–deficient CALM mutant cannot rescue increased GluA1 surface pool of CALM KO^CAG^ neurons. Surface/total SEP-GluA1 protein ratios in KO^CAG^ neurons coexpressing ubiquitin binding–deficient CALM mutant. (**D**) Tryptic synaptosome digest indicates postsynaptic HIP1/HIP1R localization. Digested synaptosomes were analyzed by immunoblotting (trypsin-treated samples normalized to untreated controls). (**E**) Coimmunoprecipitation of HIP1R-GluA1 complex from synaptosomal membranes using HIP1R-specific antibodies. (**F** and **G**) ANTH domain–containing proteins bind GluA1. GST-ANTH domains of CALM (F), HIP1, HIP1R, or GST-Epsin1-ENTH (G) were incubated with brain lysate. Analysis by immunoblotting. (**H**) HIP1/HIP1R-ANTH domains, but not Epsin1-ENTH-domain, bind ubiquitin. GST-ubiquitin or GST was incubated with His-tagged proteins. Analysis by immunoblotting. (**I**) Loss of HIP1 and HIP1R causes GluA1 surface accumulation, especially when combined with CALM deletion. Surface/total SEP-GluA1 protein ratios in neurons treated with control or HIP1- or HIP1R-specific siRNAs. (**J**) GluA1 surface accumulation upon CALM loss is rescued by overexpression of HIP1 or HIP1R. Surface/total SEP-GluA1 protein ratios in neurons transfected with pcDNA3.1 (=control), HIP1-Myc/His, or HIP1R-3xHA. (**K**) Endocytic platform of HIP1, HIP1R, and CALM fulfils overlapping cargo-specific role in endocytosis of ubiquitinated GluA1 homomers. Statistics Table 1.

Previous studies have suggested that GluA1 undergoes ubiquitination in response to synaptic activity (*48*), homeostatic scaling (*49*), (S)-3,5-dihydroxyphenylglycine (DHPG)-induced plasticity (*50*), and Aβ exposure (*13*), facilitating its endocytosis and/or subsequent sorting (*51*). Moreover, ubiquitination has been shown to underlie the endocytosis of the *C. elegans* glutamate receptor GLR-1, a process that involves the CALM/AP180-related protein UNC11 via an unknown mechanism (*52*). On the basis of these previous findings and the fact that the association of other non-ubiquitinated cargo proteins (i.e., VAMPs) with the CALM-ANTH domain is weak (*18*, *20*), we hypothesized that the CALM-mediated control of GluA1 surface levels might involve coincident detection of determinants within the cytoplasmic tail of GluA1 and of a posttranslational modification of GluA1 by ubiquitin, a process thought to involve ubiquitination of K868 (*51*) via the activity-regulated ubiquitin ligase NEDD4 (*48*). We tested this hypothesis in multiple ways: A recent in vitro study suggests that CALM and related ANTH domain proteins are able to directly associate with ubiquitin (*53*). Consistent with this proposal, we found that both, full-length CALM and its isolated ANTH domain, directly bind to GST-tagged ubiquitin immobilized on beads (Fig. 6A and fig. S7, A and B). Given these encouraging results, we compared the surface-to-internal pool of WT GluA1 to the ubiquitination-defective K868R mutant of GluA1 (*51*). Notably, interference with GluA1 ubiquitination at K868 significantly increased the pool of GluA1 on the surface of WT neurons (Fig. 6B), albeit not to the same extent as loss of CALM, consistent with a model of coincident detection of multiple determinants within GluA1 by CALM or other pathways acting in parallel (see below). Expression of ubiquitination-deficient GluA1-K868R in CALM KO^CAG^ neurons had no further effect on the surface accumulation of GluA1 (Fig. 6B). The lack of additive effects is compatible with the notion that ubiquitination and CALM-dependent internalization at mammalian synapses might act within the same pathway. Last, we used information derived from structural studies by nuclear magnetic resonance spectroscopy (*53*) to inactivate the binding site for ubiquitin within the CALM-ANTH domain by site-directed mutagenesis. We analyzed the ability of ubiquitin binding–defective CALM (CALM^Δubiquitin^) to bind GST-tagged ubiquitin immobilized on beads (Fig. 6A and fig. S7A) and to functionally rescue GluA1 surface accumulation in CALM KO^CAG^ neurons (expressed at normal levels; see figs. S6, G and H, and S7C). Ubiquitin binding–defective CALM displayed a greatly reduced ability to associate with ubiquitin in vitro (Fig. 6A and fig. S7, A and B) and was much less efficient at restoring the GluA1 surface pool to WT levels (Fig. 6C). These data show that CALM preferentially internalizes ubiquitinated GluA1 homomers by direct recognition of ubiquitin, likely in conjunction with additional sequence elements contained in the cytoplasmic tail of GluA1.

CALM is a member of a small group of endocytic proteins including HIP1 and HIP1R that harbor ANTH domains (fig. S7D) (*54*) with the propensity to recognize ubiquitin (*53*). HIP1 has previously been implicated in the endocytosis of AMPA- and NMDA-type glutamate receptors via unknown molecular mechanisms (*55*, *56*). We therefore wanted to explore whether the CALM-related ANTH domain–containing proteins HIP1 and HIP1R share overlapping functional roles with CALM in controlling GluA1 surface levels. Consistent with this hypothesis, we found HIP1 and HIP1R to reside within the postsynaptic compartment (Fig. 6D) and to associate with endogenous GluA1 in coimmunoprecipitation experiments using detergent-extracted synaptosomal membrane fractions (Fig. 6E) and in affinity chromatography experiments (Fig. 6, F and G, and fig. S7E). No interaction was detected between GluA1 and the more distantly related ENTH domain of Epsin1 or between GluA2, GluN2A, GluN2B, and the various ANTH domains (Fig. 6, F and G, and fig. S7E). The ANTH domains of HIP1 and HIP1R also bound to GST-ubiquitin (Fig. 6H and fig. S7A). Encouraged by these results, we next explored a possible overlapping role of HIP1 and HIP1R with respect to the endocytic control of GluA1 surface levels. Small interfering RNA (siRNA) mediated knockdown of either HIP1 or HIP1R (fig. S7, F and G) phenocopied CALM loss with respect to increased GluA1 surface pools (Fig. 6I). Combined deficiency of HIP1 or HIP1R and CALM further aggravated GluA1 missorting to the neuronal surface (Fig. 6I), indicating that HIP1 and HIP1R functionally overlap with CALM and may conceivably act in parallel. We tested this notion by analyzing the ability of overexpressed HIP1 or HIP1R to rescue CALM deficiency. Overexpression of either HIP1 or HIP1R (fig. S7, H and I) significantly ameliorated GluA1 accumulation on the surface of CALM KO^CAG^ neurons (Fig. 6J). Collectively, these findings demonstrate that CALM and related ANTH domain–containing proteins endocytose ubiquitinated GluA1 homomers by direct recognition of ubiquitin to control GluA1 surface pools (Fig. 6K) and thereby bidirectionally regulate postsynaptic plasticity.

## DISCUSSION

Our findings show that the ubiquitin-binding postsynaptic endocytic adaptor CALM and the related ANTH domain–containing proteins HIP1 and HIP1R act as an endocytic platform that controls the synaptic surface levels of GluA1-homomeric CP-AMPARs to antagonistically control postsynaptic LTP and LTD and, thereby, modulates spatial learning. This model (Fig. 6K) is supported by multiple independent lines of evidence: (i) We show that CALM is enriched at postsynaptic endocytic sites from which nonsynaptic AMPARs are internalized. (ii) We find that loss of neuronal CALM facilitates LTP and impairs LTD, resulting in improved spatial learning in a hippocampus-dependent paradigm. (iii) Moreover, we demonstrate that facilitation of LTP and partial occlusion of LTD in the absence of CALM are a direct consequence of elevated surface levels of functional CP-AMPARs. (iv) By combining optical imaging experiments with functional molecular analyses, we show that neuronal CALM bidirectionally controls long-term plasticity by selectively facilitating the endocytosis of GluA1 homomers via direct recognition of determinants within the GluA1 cytoplasmic domain and its site-specific modification at K868 by ubiquitin. (v) Our structure-function analyses demonstrate that this activity of CALM depends on its ability to directly bind to ubiquitinated GluA1 and to remodel membranes to facilitate endocytic vesicle formation. (vi) Last, we provide evidence that the CALM-related ANTH domain–containing endocytic proteins HIP1, a factor previously associated with AMPAR endocytosis (*55*), and its close cousin HIP1R functionally overlap with CALM, thereby defining an endocytic platform that sets and controls the number and activity of CP-AMPARs to bidirectionally direct long-term plasticity in the mammalian brain. These findings show that although CP-AMPARs constitute a small fraction of the total AMPAR surface pool, the accumulation of these high-conductance receptors can have a large impact on synaptic strength. Our data thus significantly extend the concept of an AMPAR code for synaptic plasticity (*1*) by providing a molecular basis for the subunit-specific control of GluA1 homomer surface levels. Whether and to what extent CALM affects GluA1-containing heteromers and/or their associated factors (*57*) remains an interesting subject for future studies.

Our results argue against a simplistic model according to which LTD depends only on GluA2 removal via clathrin and clathrin adaptors, while LTP is mediated by the delivery of GluA1-homomeric AMPARs (*1*). Instead, our data favor a scenario in which subunit-selective mechanisms operate to set synaptic strength in both LTP and LTD by controlling the endocytic removal of CP-AMPARs from the postsynaptic plasma membrane. The CALM-dependent endocytic pathway for CP-AMPAR internalization identified here is distinct from previously described mechanisms for the endocytosis of GluA2-containing heteromeric CI-AMPARs that use clathrin and the clathrin-associated adaptor complex AP-2 (*22*–*24*). In contrast, we observe that clathrin itself as well as its association with the clathrin adaptor CALM are dispensable for the endocytic control of GluA1 surface partitioning and internalization. It thus appears that neurons exploit clathrin-independent mechanisms of endocytosis both at the presynapse to internalize synaptic vesicle membranes (*44*, *45*) and in postsynaptic neurons [see, e.g., (*58*) for evidence for clathrin- and dynamin-independent endocytic mechanisms] to control long-term plasticity by dynamically setting the surface levels of CP-AMPARs. The notable ability of neurons to rapidly internalize membrane at pre- and postsynaptic endocytic sites may relate to unique mechanisms of Ca^2+^-triggered fission by neuron-enriched isoforms of dynamin (i.e., dynamin 1) that are activated by calcineurin (*59*) and manipulate the plasma membrane via sequence elements in their lipid-binding pleckstrin homology domains (*60*).

The existence of specific endocytic mechanisms that control the surface levels of CP-AMPARs versus CI-AMPARs at hippocampal synapses is further underscored by recent data suggesting a role for the endocytic protein PICK1 in regulating the endocytosis (*25*, *61*) and intracellular retention (*62*) of CI-AMPARs and by the observation that defective endocytosis of CI-AMPARs in the absence of synaptotagmin 3 occludes LTP decay but does not cause elevated LTP (*63*). These data together with our findings suggest that the subunit-selective endocytosis of GluA1-homomeric CP-AMPARs contributes to setting the amplitude of LTP, while the endocytic removal of GluA2-containing CI-AMPARs underlies the kinetic control of LTP decline. As endocytosis of GluA1 has also been linked to synaptic scaling (*64*), it seems likely that the CALM-mediated endocytic control of CP-AMPAR function may also be of importance for synaptic scaling during homeostatic plasticity, a prediction that should be tested in future studies.

The CALM-dependent mechanism for the subunit-selective endocytosis of CP-AMPARs at hippocampal synapses is evolutionary conserved and shares key molecular features with the internalization of the AMPA-related GLR-1 receptor at *C. elegans* synapses that is mediated by the single CALM/AP180 ortholog in worms (*52*). An important characteristic is its dependence on ubiquitin, which acts as molecular signpost for endocytosis and, possibly, for downstream endosomal sorting of ubiquitinated GluA1 to prevent their immediate exocytic return to the neuronal surface. Our data thus support a crucial role for AMPAR ubiquitination in the control of Hebbian plasticity.

The function of CALM in the subunit-selective endocytosis of ubiquitinated CP-AMPARs at central synapses is partially shared with the ANTH domain–containing proteins HIP1 and HIP1R. While HIP1R has not been implicated in synaptic plasticity or the endocytosis of postsynaptic glutamate receptors before, mice lacking HIP1 display reduced LTD and suffer from progressive neurological defects culminating in tremor, gait ataxia, and premature death (*55*). These severe phenotypes likely are a reflection of the more pleiotropic roles of HIP1 and HIP1R (*65*) in the mammalian brain, for example, in the regulation of actin polymerization and NMDAR function (*56*). In our in vivo models, the severity of CALM loss is likely ameliorated by the presence of HIP1 and HIP1R. It would thus be interesting to analyze LTD and CP-AMPAR endocytosis in neurons from CALM/HIP1/HIP1R triple-deficient mice. Together, our results are most compatible with a model according to which CALM and related ANTH domain–containing proteins, possibly in conjunction with the ubiquitin-binding scaffold Eps15 (*66*), act as a clathrin-independent endocytic platform that tunes the surface levels of CP-AMPARs to shape synaptic plasticity. The precise nanoscale localization of CP-AMPAR endocytosis events and their effects on synaptic versus extrasynaptic CP-AMPAR pools (*67*) remain important subjects for future studies. On the basis of our own data and those of others (*68*, *69*), we consider it likely that CALM-mediated endocytosis of GluA1-containing AMPARs occurs at perisynaptic sites and may thereby alter the levels of GluA1-containing AMPARs at extrasynaptic sites and at synapses.

Last, it is intriguing to consider how CALM might contribute to the mechanisms that underlie memory decline in AD. Variants of *PICALM*, the gene encoding CALM, are strongly linked to AD (*16*) via diverse proposed mechanisms that range from the regulation of autophagy (*70*) and γ-secretase trafficking (*17*) to Aβ clearance by brain endothelial cells (*71*). Our data suggest an alternative possibility: We hypothesize that neuronal CALM is at the heart of an Aβ-induced mechanism that promotes the NEDD4-1–mediated ubiquitination (*72*), endocytosis, and degradation of GluA1-containing AMPARs (*14*) to induce sustained and irreversible synaptic depression. This idea is consistent with a recent study suggesting that Aβ causes synaptic dysfunction by creating a metaplastic state that favors LTD signaling (*15*). An important contribution of GluA1 to memory decline in AD is also supported by the fact that non-amyloidogenic cleavage of amyloid precursor protein (APP) by γ-secretase promotes LTP and counteracts memory decline by increasing the exocytic delivery and, thereby, the synaptic levels of CP-AMPARs (*73*), akin to the effects observed upon decreased expression of CALM in our functional electrophysiological experiments (see Fig. 2). CALM may thus function as a synaptic homeostat that sets the levels of CP-AMPARs to control synaptic weight under physiological (e.g., learning) and pathological conditions including AD, excitotoxic cell death (*74*), and epilepsy. Pharmacological targeting of CALM and/or its association with GluA1 homomers could thus serve as an innovative strategy to combat aging-related memory decline and AD.

## MATERIALS AND METHODS

### Experimental design

Sample sizes were not predetermined using power analysis, since they were not chosen on the basis of prespecified effect size. Instead, multiple independent experiments were carried out using several biological replicates. Detailed descriptions of sample size and statistical analysis used to test normality and calculate *P* values are given in Table 1, in supplementary figure legends, and in the “Statistical analysis” section. Data were collected and analyzed by multiple researchers, blind to the conditions whenever experimentally possible. All experiments were performed in a controlled laboratory setting.

**Table 1.** Overview statistics.

**Figure**	**Data type**	**Sample size (*N*, *n*) and *P* value**	**Statistical test**
1C	Mean ± SEM	*N* = independent experiments	One-sample *t* test
		CALM: *N* = 7, *P* = 0.0002	
		AP180: *N* = 5, *P* = 0.5840	
		GluN2A: *N* = 5, *P* = 0.0001	
		GluN2B: *N* = 4, *P* = 0.0008	
		GluA1: *N* = 7, *P* ≤ 0.0001	
		GluA2: *N* = 4, *P* ≤ 0.0001	
		Syp: *N* = 3, *P* = 0.3311	
		SNAP-25: *N* = 4, *P* = 0.0918	
		Rab3a: *N* = 6, *P* = 0.1094	
		Endo A1: *N* = 4, *P* = 0.1023	
		Clathrin: *N* = 4, *P* = 0.0771	
		AP-2α: *N* = 4, *P* = 0.8047	
		AP-2 μ: *N* = 4, *P* = 0.3059	
		FCHo2: *N* = 4, *P* = 0.3560	
2A	Mean ± SEM	CALM^Tub^: *N* = 21 animals, *P* ≤ 0.0001	One-sample *t* test
		CALM^EMX^: *N* = 8 animals, *P* ≤ 0.0001	
2C	Mean ± SEM	WT: *N* = 5 animals, *n* = 12 slices	Two-tailed unpaired *t* test
		CALM KO^EMX^: *N* = 5 animals, *n* = 12 slices, *P* = 0.0027	
2E	Mean ± SEM	WT: *N* = 5 animals, *n* = 10 slices	Two-tailed unpaired *t* test
		CALM KO^EMX^: *N* = 5 animals, *n* = 10 slices, *P* ≤ 0.0001	
2G	Mean ± SEM	WT: *N* = 7 animals, *n* = 13 slices	Two-tailed unpaired *t* test
		CALM KO^EMX^: *N* = 7 animals, *n* = 13 slices, *P* = 0.0384	
2 (H to J)	Mean ± SEM	WT: *N* = 16 animals	Mann-Whitney test
		CALM KO^EMX^: *N* = 18 animals	
		Escape latency day 2: *P* = 0.0054	
		Escape latency day 3: *P* = 0.0184	
3 (B and C)	Mean ± SEM	*N* = number of independent experiments	One-sample *t* test
		GluA1: *N* = 3, *P* = 0.0365	
		GluA2: *N* = 3, *P* = 0.1873	
3E	Mean ± SEM	*N* = 3 independent organotypic cultures with *n* at least 6 uncaging spots from 9 WT and 8 CALM^EMX^ neurons; *P* = 0.0008	Two-tailed unpaired *t* test
3F	Mean ± SEM	*N* = 3 independent organotypic cultures with *n* at least 13 uncaging spots from 12 control and 11 CALM^KD^ neurons; *P* = 0.0043	Two-tailed unpaired *t* test
3G	Mean ± SEM	*N* = 3 independent organotypic cultures transduced with pAAV-CALM mCherry with *n* at least 4 uncaging spots from 8 CALM^EMX^, control, and 4 CALM^EMX^ neurons; *P* = 0.0010	Two-tailed unpaired *t* test
3 (H and I)	Mean ± SEM	WT: *N* = 4 animals, *n* = 10 slices	Two-tailed unpaired *t* test
		CALM KO^EMX^: *N* = 4 animals, *n* = 10 slices, *P* = 0.3631	
3 (J and K)	Mean ± SEM	WT: *N* = 6 animals, *n* = 12 slices	Two-tailed unpaired *t* test
		CALM KO^EMX^: *N* = 6 animals, *n* = 12 slices, *P* = 0.5734	
3 (L to N)	Mean ± SEM	Sham	One-way ANOVA with Holm-Sidak method
		WT: *N* = 5 animals, *n* = 7 slices	
		CALM KO^EMX^: *N* = 5 animals, *n* = 6 slices	
		IEM 1460	
		WT: *N* = 5 animals, *n* = 8 slices	
		CALM KO^EMX^: *N* = 5 animals, *n* = 8 slices	
		Sham(WT) versus Sham(CALM^EMX^): *P* = 0.0003	
		Sham(CALM^EMX^) versus IEM 1460(CALM^EMX^): *P* = 0.0001	
4A	Mean ± SEM	*N* = 5 independent experiments, *P* ≤ 0.0001	Two-tailed unpaired *t* test
4B	Mean ± SEM	*N* = 4 independent experiments, *P* = 0.8328	Two-tailed unpaired *t* test
4C	Mean ± SEM	*N* = 4 independent experiments	One-way ANOVA followed by Dunnett’s post hoc test
		iRFP(WT) versus iRFP(CALM^CAG^): *P* = 0.0009	
		iRFP(WT) versus CALM WT(CALM^CAG^): *P* = 0.4672	
4E	Mean ± SEM	*N* = independent experiments	One-sample *t* test
		GluA1: *N* = 9, *P* = 0.0053	
		GluA2: *N* = 6, *P* = 0.3893	
		GluN2B: *N* = 4, *P* = 0.8245	
		N-cadherin: *N* = 7, *P* = 0.5010	
4F	Mean ± SEM	*N* = 4 independent experiments	One-way ANOVA followed by Dunnett’s post hoc test
		A1 + A1(WT) versus A1 + A1(CALM^CAG^): *P* = 0.0005	
		A1 + A2(WT) versus A1 + A2(CALM^CAG^): *P* = 0.4922	
		A2 + A2(WT) versus A2 + A2(CALM^CAG^): *P* = 0.9999	
4I	Mean ± SEM	*N* = 3 independent experiments	One-way ANOVA followed by Dunnett’s post hoc test
		Synaptic(WT) versus synaptic (CALM^CAG^): *P* = 0.4653	
		Extrasynaptic(WT) versus extrasynaptic(CALM^CAG^): *P* = 0.3618	
		Synaptic(WT) versus extrasynaptic(WT): *P* ≤ 0.0001	
		Synaptic(CALM^CAG^) versus extrasynaptic(CALM^CAG^): *P* ≤ 0.0001	
4K	Mean ± SEM	*N* = 4 independent experiments, *P* = 0.0423	One-sample *t* test
4M	Mean ± SEM	*N* = 3 independent experiments, *P* = 0.3417	One-sample *t* test
5B	Mean ± SEM	*N* = 15 cells, *n* = 464 events	No testing
5E	Mean ± SEM	*N* = 4 independent experiments, *P* = 0.5990	Two-tailed unpaired *t* test
5G	Mean ± SEM	*N* = 3 independent experiments	One-way ANOVA followed by Dunnett’s post hoc test
		iRFP(WT) versus iRFP(CALM^CAG^): *P* ≤ 0.0001	
		iRFP(WT) versus WT(CALM^CAG^): *P* = 0.5847	
		iRFP(WT) versus ΔPIP2(CALM^CAG^): *P* ≤ 0.0001	
		iRFP(WT) versus ΔH0(CALM^CAG^): *P* ≤ 0.0001	
		iRFP(WT) versus Δcargo(CALM^CAG^): *P* ≤ 0.0001	
		iRFP(WT) versus Δclathrin(CALM^CAG^): *P* = 0.9056	
5H	Mean ± SEM	*N* = 10 independent experiments, *P* ≤ 0.0001	One-sample *t* test
5J	Mean ± SEM	*N* = 3 independent experiments, *P* = 0.0009	One-sample *t* test
5K	Mean ± SEM	*N* = 3 independent experiments, *P* = 0.0169	Two-tailed unpaired *t* test
5L	Mean ± SEM	*N* = 3 independent experiments, *P* = 0.8754	Two-tailed unpaired *t* test
6B	Mean ± SEM	*N* = 3 independent experiments	One-way ANOVA followed by Dunnett’s post hoc test
		GluA1 WT(WT) versus GluA1 WT(CALM^CAG^): *P* = 0.0004	
		GluA1 WT(CALM^CAG^) versus GluA1 K868R(WT): *P* = 0.1017	
		GluA1 WT(CALM^CAG^) versus GluA1 K868R(CALM^CAG^): *P* = 0.3623	
6C	Mean ± SEM	*N* = 4 independent experiments, *P* = 0.0008	One-way ANOVA followed by Dunnett’s post hoc test
6D	Mean ± SEM	*N* = 5 independent experiments	One-sample *t* test
		HIP1: *P* = 0.0040	
		HIP1R: *P* = 0.0039	
6I	Mean ± SEM	*N* = independent experiments	One-way ANOVA followed by Dunnett’s post hoc test
		siCTRL(WT): *N* = 6	
		siCTRL(CALM^CAG^): *N* = 6	
		siHIP1(WT): *N* = 4	
		siHIP1(CALM^CAG^): *N* = 4	
		siHIP1R(WT): *N* = 4	
		siHIP1R(CALM^CAG^): *N* = 4	
		siCTRL(WT) versus siCTRL(CALM^CAG^): *P* ≤ 0.0001	
		siCTRL(WT) versus siHIP1(WT): *P* = 0.0010	
		siCTRL(WT) versus siHIP1(CALM^CAG^): *P* ≤ 0.0001	
		siCTRL(WT) versus siHIP1R(WT): *P* = 0.0003	
		siCTRL(WT) versus siHIP1R(CALM^CAG^): *P* ≤ 0.0001	
		siCTRL(CALM^CAG^) versus siHIP1(CALM^CAG^): *P* = 0.0197	
		siCTRL(CALM^CAG^) versus siHIP1R(CALM^CAG^): *P* = 0.0190	
6J	Mean ± SEM	*N* = 3 independent experiments	One-way ANOVA followed by Dunnett’s post hoc test
		pcDNA3.1(WT) versus pcDNA3.1(CALM^CAG^): *P* ≤ 0.0001	
		pcDNA3.1(CALM^CAG^) versus HIP1-myc/his (CALM^CAG^): *P* = 0.0007	
		pcDNA3.1(CALM^CAG^) versus HIP1R-3xHA(CALM^CAG^): *P* = 0.0051	

### Animals

All animal experiments involving mice were reviewed and approved by the ethics committee of the “Landesamt für Gesundheit und Soziales” (LAGeSo) Berlin and were conducted according to the committee’s guidelines under animal experimentation permits T0243/08, S0313/17, G0341/14, G0040/16, and G0323/18. At the institute, the Animal Care Officer and the LAGeSo monitored compliance with all regulations. The mice were looked after by professional caretakers and checked daily. All animals except for AP180 KO mice, which have been described before (*28*) (line name: Snap91^tm1.1Tmar^), have a normal health and immune status. AP180 KO mice suffer from epileptic seizures and premature death as described in (*28*). The animal facility where the mice are kept is regularly checked for standard pathogens. Mice from both genders were used. The age of the used animals is reported in the respective experimental sections. Mice were kept in groups of up to six animals in standard individually ventilated cages (IVC) cages of 524 cm^2^ containing bedding and nesting material. Food and water were provided ad libitum. The light cycle ran from 6 a.m. to 6 p.m.

### Generation of tissue-specific and inducible PICALM KO mouse lines

PICALM lox/lox mice were provided by T. Maeda and are described in (*75*). These mice were crossed with three different Cre recombinase driver lines to obtain tissue-specific or inducible PICALM KO mice: (i) with EMX1-Cre mice [described in (*30*)] to generate forebrain-specific PICALM KO mice, (ii) with Tubulin α1-Cre mice [described in (*29*)] to generate pan-neuronal PICALM KO mice, and (iii) with B6.Cg-Tg(CAG-cre/Esr1*)5Amc/J mice obtained from The Jackson Laboratory [stock number: 004682; described in (*38*)] to create tamoxifen-inducible PICALM KO neurons in culture. Throughout the text, PICALM lox/lox cre-negative mice are denoted as “WT,” PICALM lox/lox EMX1 cre-positive mice as “CALM^EMX^,” PICALM lox/lox tubulin α-1 cre-positive mice as “CALM^Tub^,^”^ and tamoxifen-treated PICALM lox/lox iCAG cre-positive cells as “CALM^CAG^.”

### Behavioral experiments

Behavioral experiments were performed during the light cycle. For the elevated plus maze, mice were placed at the center of the apparatus (Ugo Basile, #40143) facing a closed arm. Their behavior was recorded for 5 min. To be counted as an entry, the mouse had to enter an arm with all four paws. For the Morris water maze task, two independent cohorts of 6- to 7-month-old mice (first cohort: 8 WT, 4 males and 4 females, and 8 CALM^EMX^, 4 males and 4 females; second cohort: 8 WT, 4 males and 4 females, and 10 CALM^EMX^, 4 males and 6 females) were transferred to the Preclinical Research Center of the Max-Delbrück-Center for Molecular Medicine 2 weeks before the experiments for habituation. The water maze consisted of a circular pool (diameter, 150 cm) filled with water (21° ± 1°C). On the first day (probing phase), the mice were trained to locate a visible platform (diameter, 15 cm) located approximately 0.5 cm above the water level and indicated with a visible flag, forcing the mice to use distal cues for spatial orientation. Four shapes around the pool (lightning, triangle, square, and cross) served as visual cues. After the visible platform test was completed, the mice were trained for the next 5 days to locate a hidden platform (1 cm below water level) with four training trials per day (with 1-hour intertrial interval). Each trial began by placing the mouse into the water, near and facing the wall of the pool. The position of pool entry from four different directions was shuffled daily. Each subject was allowed 90 s to find the platform, and if it failed to reach the platform within 90 s, it was guided by the experimenter to the platform and allowed to stay on the platform for 20 s. The mice that did not reach the platform during a trial were assigned a latency of 90 s. For the probe test on the last day of training, the platform was removed, and mice were placed into the pool near the wall in the quadrant opposite to that of the previous platform location and allowed to search for the platform for 1 min. Upon removal from the maze, the mice were dried with absorbent paper and returned to their home cages. Swim paths were recorded by an overhead camera, and parameters such as swim speed and latency to reach the platform were extracted.

### Materials

The following materials were used in this study: (*Z*)-4 hydroxytamoxifen (Sigma-Aldrich, catalog no. H7904), glycine (Sigma-Aldrich, catalog no. G7403), GST-ubiquitin (Boston Biochem, catalog no. U-540), IEM 1460 (Tocris, catalog no. 1636), NMDA (Sigma-Aldrich, catalog no. M3262), tetrodotoxin (TTX; Tocris Bioscience, catalog no. 1078), picrotoxin (Sigma, catalog no. P1675), DL-AP5 (Tocris, catalog no.0105), NBQX disodium salt (Tocris, catalog no.1044/1), transferrin from human serum, Alexa Fluor 647 conjugate (Thermo Fisher Scientific, catalog no. T23366), EZ-Link Sulfo-NHS-LC-Biotin (Thermo Fisher Scientific, catalog no. 21335), GST•Bind Resin (Merck, catalog no. 70541), HIS-Select Nickel Affinity Gel (Merck, catalog no. P6611), Phusion Site-Directed Mutagenesis Kit with DH10B Competent Cells (Thermo Fisher Scientific, catalog no. F542), Pierce BCA Protein Assay Kit (Thermo Fisher Scientific, catalog no. 23225), Pierce Protein A/G Magnetic Beads (Thermo Fisher Scientific, catalog no. 88803), ProFection Mammalian Transfection System (Promega, catalog no. E1200), streptavidin agarose (Merck, catalog no. 69203), ON-TARGETplus Mouse Hip1 siRNA (Dharmacon, catalog no. J-053463-05), ON-TARGETplus Mouse Hip1r siRNA (Dharmacon, catalog no. J-059792-10), and ON-TARGETplus Non-targeting siRNA (Dharmacon, catalog no. D-001810-01-20).

### Oligonucleotides

For genotyping, genomic DNA was extracted from ear, tail, or phalanx biopsies and analyzed with a standard polymerase chain reaction (PCR) program using the primers indicated as follows: AP180 KO line: 2F3 (CCAGATGACCTGAGTTTGTG), DL1 (TCTGGTGGATAGTGTCACTTAGGTA), and Lox1 (ACCTCATGTGAAACGTTGCCTG) (KO PCR: 2F3 × DL1; WT PCR: DL1 × Lox1); PICALM LOX line: TM435 (GTGGGATGAATGGTTGGGCTTC) and TM436 (CCATGTAGGGTCTAAAGATC); EMX Cre and CAG Cre lines: TM 63 (CCGGGCTGCCACGACCAA) and TM 64 (GGCGCGGCAACACCATTTTT); Tubulin α1-Cre line: Cre 1 (ACCTGAAGATGTTCGCGATTATCT) and Cre 2 (ACCGTCAGTACGTGAGATACTTT).

### Acute slice preparation, fEPSP, and whole-cell patch clamp recordings

Mice were quickly decapitated after cervical dislocation, and the brains were extracted into ice-cold dissection artificial cerebrospinal fluid (ACSF) containing the following: 2.5 mM KCl, 1.25 mM NaH_2_PO_4_, 24 mM NaHCO_3_, 1.5 mM MgSO_4_, 2 mM CaCl_2_, 25 mM glucose, and 250 mM sucrose. The brain was cut along the middle line, and sagittal slices (350 μm thick) were prepared from both hemispheres using a vibroslicer (Leica, VT 1200S). Slices were prepared in preoxygenated and continuously bubbled (95% O_2_/5% CO_2_) dissection ACSF at low temperature (4°C) and collected in a resting chamber containing ACSF in which 250 mM sucrose was replaced with 120 mM NaCl (pH 7.35 to 7.4). The slices were left to recover in the resting solution, continuously oxygenated at room temperature (22° to 24°C), for at least 1.5 hours before recordings. After recovery, slices were transferred into a submerged recording chamber (Warner Instruments, RC-27L), filled with ACSF with a solution exchange of 3 to 5 ml/min at 22° to 24°C. An upright microscope (Olympus, BX61WI) was used for the positioning of slices to have access to the CA1 region of the hippocampus for electrode placement. The electrodes were prepared from glass capillaries (Hilgenberg) using micropipette puller Sutter P-1000 (Sutter Instruments). Stimulating (1 to 1.5 megohms) and recording (1.5 to 2.5 megohms) electrodes filled with ACSF were placed in stratum radiatum of the CA1 region, and fEPSPs were recorded. The data were recorded at a sampling rate of 10 kHz, low-pass–filtered at 3 kHz, and analyzed using PATCHMASTER software (HEKA Elektronik). Synaptic transmission and plasticity were analyzed at the Schaffer collateral pathway. Stimulating and recording electrodes were placed in a visually preselected area of CA1 stratum radiatum and slowly advanced until maximal fEPSPs were obtained. Basal stimulation of 0.2-ms electrical pulses was delivered at 0.05 Hz at the stimulation intensity, which induced approximately 30 to 50% of the maximal responses. After 10 min of stable baseline recordings, input/output stimulus response curves were made as a measure of basal excitatory synaptic transmission. Stimulation intensity was increased by 20-μA steps until the maximal fEPSP was obtained, defined as a response with superimposed population spike component on decreasing fEPSP trace. The slopes of fEPSPs were plotted versus presynaptic fiber volleys as a function of increasing stimulation intensity. A PPF protocol was used to test short-term presynaptic plasticity, and facilitation was calculated as a percentage increase of the slope of the second response as compared to the first. Two pulses at time intervals 10, 20, 50, 100, 200, and 500 ms were delivered at a stimulation intensity, which induced one-third of the maximal responses. For short intervals (10 and 20 ms), the first fEPSPs were digitally subtracted before measurements of the second. Each value measured is an average of three consecutive stimulations repeated every 20 s for stimulus responses and every 30 s for PPF measurements. To test the role of CALM deficiency in synaptic plasticity, we induced LTP and LTD. For these experiments, the stimulation intensity was selected to elicit 50% of its maximum amplitude, and basal stimulation was monitored at 0.05 Hz. LTP was induced using single theta-burst stimuli (TBS) containing eight bursts at 5 Hz, each burst containing four pulses at 100 Hz. LTD was induced using an LFS protocol, which consisted of 900 pulses at 1 Hz, and stimulation intensity was set to 100% of its maximum during 1-Hz stimulation. LTP and LTD were recorded for 1 hour after their induction and calculated as percent increase or decrease of the fEPSP slope between 50 and 60 min as compared to the initial 10 min of baseline. In all figures, each value is an average of three consecutive time points recorded every 20 s, and the mean slopes of the fEPSPs recorded 0 to 10 min before 1× TBS or LFS are taken as 100%. The CP-AMPAR antagonist IEM 1460 (50 μM) was applied to the bath either immediately or 30 min after LFS. NMDA/AMPA ratios were studied by isolating NMDAR-mediated fEPSPs and comparing them to initial AMPA responses. Stimulation intensity was set to 60 to 70% of the maximum responses, and stimulation occurred every 20 s in the presence of AMPA/kainate receptor antagonist NBQX (10 μM) and γ-aminobutyric acid type A (GABA_A_)/glycine receptor antagonist picrotoxin (50 μM) and reduced extracellular Mg^2+^ ion concentration (from 1.5 to 0.25 mM). CA3-CA1 connections were dissected to avoid epileptic-like discharges induced by combination of GABAergic antagonist and afferent stimulation. Stabile NMDAR-mediated responses were isolated for up to 50 min and compared to initial AMPAR-mediated responses, which were taken as 100%. At the end of every experiment, the potent NMDAR antagonist d,l-2-amino-5-phosphonovaleric acid (APV; 50 μM) was applied to verify NMDA responses. APV (50 μM) reduced the responses further to about 2% of the initial values. The amplitudes of AMPAR and NMDAR responses were measured in their maximal peak area and plotted as NMDA/AMPA ratios. NMDA/AMPA ratios were calculated by dividing average amplitudes of NMDAR-mediated responses between 40 and 50 min by the initial 10 min of AMPAR-mediated responses. The remaining APV-insensitive (about 2%) responses, mostly reflecting noise, were subtracted from both NMDA and AMPA values before calculating NMDA/AMPA ratios. Whole-cell patch clamp recordings from CA1 principal cells upon Schaffer collateral stimulation (to estimate the rectification index) were performed with the same conditions used for fEPSPs recordings, except that the whole-cell modality was achieved with glass capillaries (4 to 8 megohms) filled with intracellular solution containing the following: 135 mM K·CH_3_SO_3_, 4 mM NaCl, 2 mM MgCl_2_, 2 mM Na_2_ATP, 0.3 mM Na_2_GTP, 0.06 mM EGTA, 0.01 mM CaCl_2_, and 10 mM HEPES, adjusted to 300 to 315 mOsm/liter and pH 7.2 to 7.3. SigmaPlot (Systat) software was used for data analyses and presentation. Data were statistically evaluated using two-way analysis of variance (ANOVA) with repeated measures. For comparisons of two groups, statistical significance was tested by two-tailed unpaired Student’s *t* test. Values are depicted as means ± SEM. *n* and *N* indicate the number of tested slices and mice, respectively.

### DNA constructs

The following expression vectors were used in this study and were obtained from the indicated sources: HA-GluA1 and HA-GluA2 (gift from Y. T. Wang); HA-SEP-GluA1, myc-SEP-GluA2, pmCherry-N1-CALM, and TrfR-pHuji (gift from D. Perrais); HIP1-myc/his and HIP1R-3xHA (gift from T. S. Ross); pcDNA3.1_nHA spinophilin (Addgene, 87122); pET28a(+) His-CALM ANTH WT, pET28a(+) His-CALM ANTH Δcargo, and pGEX-4T1 CALM ANTH [described in (*18*)]; pGEX-4T1 GluA1 C-terminal [described in (*22*)]; and synaptophysin-pHluorin (gift from L. Lagnado). The different *Mus musculus* CALM-deficient mutants used in this study (ΔPIP_2_, ΔH_0_, Δcargo, Δclathrin, and Δubiquitin) were synthetized by Absea Biotechnology Ltd. and inserted into a pGex4T-1 vector between Bam HI and Xho I. For rescue experiments, they were subcloned via Bam HI/Xho I into the backbone vector pAAV-CALM WT (provided by J. M. Lee) containing a C-terminal HA tag. His-CALM ANTH ubiquitin mutant (Δubiquitin) was subcloned by PCR using the construct synthesized by Absea Biotechnology Ltd. and inserted between Bam HI and Not I into pET-28a(+). The complementary DNA (cDNA) encoding for the WT full-length *M. musculus* C-terminal tagged CALM mCherry, Δclathrin, and Δubiquitin was subcloned into the backbone vector pAAV-CMV-iRFP (Addgene, #64887) via Cla I/Sal I. HA-SEP-GluA1 K866R was generated via direct mutagenesis using the Phusion Site-Directed Mutagenesis Kit (Thermo Fisher Scientific) from HA-SEP-GluA1 WT. For bacterial expression, the HIP1 ANTH domain (*Homo sapiens*, amino acids 1 to 314), the HIP1R ANTH domain (*H. sapiens*, amino acids 1 to 305), and the Epsin1 ENTH domain (*Rattus norvegicus*, amino acids 1 to 164) were subcloned into pGEX-4T1 and pET-28a(+). Spinophilin-mCherry was generated by introducing the full-length cDNA encoding *H. sapiens* spinophilin (Addgene, #87122) into the backbone vector pmCherry-C1 via Hind III/Sal I. All PCR-amplified DNAs were confirmed by DNA sequence analyses. Plasmids were amplified in bacteria (*Escherichia coli*, TOP10), and endotoxin-free DNA preparations were used according to standard procedures. All plasmids used in the study are available from corresponding authors without restriction.

### Antibodies

The following primary antibodies were used in this study. Mouse antibodies used were against the following: AP-2μ (BD Biosciences, catalog no. 611351; RRID:AB_398873), AP-2α (BD Biosciences, catalog no. 610502; RRID:AB_397868), CALM (Santa Cruz Biotechnology, catalog no. sc-271224; RRID:AB_10611476), clathrin heavy chain (CHC; V. Haucke laboratory; clone TD1), Eps15 (gift from S. Sigismund; clone 15-3T), GluA1 (Millipore, catalog no. MAB2263; RRID:AB_1977459), GluA2 (Millipore, catalog no. MAB397; RRID:AB_11212990), HIP1R (Santa Cruz Biotechnology, catalog no. sc-135937; RRID:AB_2117577), His tag (Millipore, catalog no.70796-3; RRID:AB_10807496), Homer 1 (Synaptic Systems, catalog no. 160 011; RRID:AB_2120992), N-cadherin (BD Biosciences, catalog no. 610920; RRID:AB_2077527), PSD95 (Synaptic Systems, catalog no. 124 011BT; RRID:AB_2619799), Rab3a (Synaptic Systems, catalog no. 107 111; RRID:AB_887770), SNAP-25 (Synaptic Systems, catalog no. 111 011; RRID:AB_887794), synaptophysin 1 (Synaptic Systems, catalog no. 101 011C3; RRID:AB_887822), syntaxin 1 (Synaptic Systems, catalog no. 110 111; RRID:AB_887848), and β-actin (Sigma-Aldrich, catalog no. A5441; RRID:AB_476744). Rabbit antibodies used were against the following: anti-GFP (Abcam, catalog no. ab6556; RRID:AB_305564), AP180 (Synaptic System, catalog no. 155 003; RRID:AB_887691), CALM (Novus Biologicals, catalog no. NBP1-86658; RRID:AB_11024511), CALM (Sigma-Aldrich, catalog no. HPA019061; RRID:AB_1855362), Living Colors DsRed (Takara Bio, catalog no. 632496; RRID:AB_10013483), endophilin A1 (Synaptic System, catalog no. 159 002; RRID:AB_887757), Eps15R (gift from S. Sigismund, clone si114), FCHO2 (Thermo Fisher Scientific, catalog no. PA5-31696; RRID:AB_2549169), GluA1 pSer^845^ (Cell Signaling Technology, catalog no. 8084; RRID:AB_10860773), GluA3 (Cell Signaling Technology, catalog no. 4676; RRID:AB_10547136), GluA4 (Cell Signaling Technology, catalog no. 8070; RRID:AB_10829469), GluN2A (Cell Signaling Technology, catalog no. 4205; RRID:AB_2112295), GluN2B (Cell Signaling Technology, catalog no. 4207; RRID:AB_1264223), GST tag (Thermo Fisher Scientific, catalog no. 71-7500; RRID:AB_2533994), HA tag (GeneTex, catalog no. GTX115044; RRID:AB_10622369), HIP1 (Abcam, catalog no. ab181238), HIP1R (GeneTex, GTX65566), and Myc tag (Cell Signaling Technology, catalog no. 2272; RRID:AB_10692100). Guinea pig antibodies used were against the following: MAP 2 (Synaptic System, catalog no. 188004; RRID:AB_2138181) and Bassoon (Synaptic Systems, catalog no. 141 004; RRID:AB_2290619). The following secondary antibodies were used in this study: Alexa Fluor 488 AffiniPure Donkey Anti-Guinea Pig IgG (H+L) Jackson ImmunoResearch (catalog no. 706-545-148; RRID:AB_2617153), ATTO 647N (STED/GSD) Goat Anti-Rabbit IgG Active Motif (catalog no. 15048), Goat Anti-Guinea Pig IgG (H+L) Highly Cross-Adsorbed Secondary Antibody, Alexa Fluor 647 (Thermo Fisher Scientific, catalog no. A-21450; RRID:AB_2735091), Goat Anti-Mouse IgG (H+L) Highly Cross-Adsorbed Secondary Antibody, Alexa Fluor 594 (Thermo Fisher Scientific, catalog no. A-11032; RRID:AB_2534091), Goat Anti-Mouse IgG (H+L) Cross-Adsorbed Secondary Antibody, Alexa Fluor 488 (Thermo Fisher Scientific, catalog no. A-11001; RRID:AB_2534069), Goat Anti-Mouse IgG (H+L) Highly Cross-Adsorbed Secondary Antibody, Alexa Fluor 647 (Thermo Fisher Scientific, catalog no. A-21236; RRID:AB_2535805), Goat Anti-Mouse IgG (H+L) Highly Cross-Adsorbed Secondary Antibody, Alexa Fluor 568 (Thermo Fisher Scientific, catalog no. A-11031; RRID:AB_144696), Goat Anti-Rabbit IgG (H+L) Cross-Adsorbed Secondary Antibody, Alexa Fluor 488 (Thermo Fisher Scientific, catalog no. A-11008; RRID:AB_143165), IRDye 680RD donkey anti-mouse IgG secondary antibody (LI-COR Biosciences, catalog no. 926-68072; RRID:AB_10953628), IRDye 680RD donkey anti-rabbit IgG secondary antibody (LI-COR Biosciences, catalog no. 926-68073; RRID:AB_10954442), IRDye 800CW donkey anti-mouse IgG secondary antibody (LI-COR Biosciences, catalog no. 926-32212; RRID:AB_621847), and IRDye 800CW donkey anti-rabbit IgG secondary antibody (LI-COR Biosciences, catalog no. 926-32213; RRID:AB_621848). For immunoprecipitation experiments, the following immunoglobulin G (IgG) isotype controls were used in this study: mouse (Thermo Fisher Scientific, catalog no. 31903; RRID:AB_10959891) and rabbit (Thermo Fisher Scientific, catalog no. 10500C; RRID:AB_2532981).

### Preparation of neuronal cell cultures and transfection

Cortico-hippocampal neurons or hippocampal neurons were isolated from neonatal mouse brains [postnatal days (p) 0 to 3] and were prepared in sparse or mass culture, respectively. Briefly, cortices and hippocampi or hippocampi alone (pooled from several genotypically identical littermates) were rapidly dissected under a binocular microscope, placed into ice-cold HEPES-buffered Hanks’ balanced salt solution (HBSS; Thermo Fisher Scientific) containing 20% fetal bovine serum (FBS), and cut with a scalpel into ca. 1-mm^3^-sized pieces. The tissue pieces were washed first with HBSS containing 20% FBS and then with HBSS only and afterwards were digested for 15 min in digestion buffer [137 mM NaCl, 5 mM KCl, 7 mM Na_2_HPO_4_, 25 mM HEPES, trypsin (1 mg/ml), and 1500 U of deoxyribonuclease (DNase) (pH 7.2)] at 37°C, followed by another washing step with HBSS and gentle trituration in dissociation buffer [HBSS containing 12 mM MgSO_4_ and 1500 U of DNase (pH 7.2)]. A total of 100,000 (for each well of a 6-well plate) or 50,000 hippocampal neurons (for each well of a 12-well plate) were plated as 40- or 20-μl drops, respectively, per poly-l-lysine–coated coverslip. A total of 2 ml (for each well of a 6-well plate) or 1 ml (for each well of a 12-well plate) of plating medium [basic medium (MEM medium) supplemented with 0.5% glucose, 0.02% NaHCO_3_, 0.01% transferrin, 10% FBS, 2 mM l-glutamine, insulin (25 μg/ml), and 1% penicillin-streptomycin] was added 1 hour after plating. For biotinylation experiments, 1,200,000 cortico-hippocampal neurons (for each well of a six-well plate) were plated as sparse culture directly in 2 ml of plating medium. After 1 day in vitro (DIV1), half of the plating medium was replaced by growth medium (basic medium containing 5% FBS, 0.5 mM l-glutamine, 1× B27 supplement, and 1% penicillin-streptomycin). On DIV2, 1 ml (for each well of a 6-well plate) or 500 μl (for each well of a 12-well plate) of growth medium was added. Cytosine b-D-arabinofuranoside (AraC) (2 μM) was used during medium renewal to limit glial proliferation. Neurons were maintained at 37°C in a 5% CO_2_ humidified incubator until DIV12 to DIV14 for cortico-hippocampal neurons or DIV14 to DIV16 for hippocampal neurons. To initiate gene deletion, primary neurons isolated from floxed animals expressing a tamoxifen-inducible Cre recombinase (CALM^CAG^) were treated with 0.3 μM (*Z*)-4-hydroxytamoxifen (Sigma-Aldrich) immediately after plating. An equal concentration of tamoxifen (0.3 μM) was used during medium renewal on DIV1 and DIV2. Neurons derived from floxed littermates that were Cre negative (WT) were used as controls and were treated with equal amounts of (*Z*)-4-hydroxytamoxifen. For transient protein expression, neurons were transfected on DIV7 to DIV8 by calcium phosphate (ProFection Mammalian Transfection System, Promega) using between 0.5 and 4 μg of plasmids, 250 mM CaCl_2_, and nuclease-free water (for each well of a six-well plate) mixed with equal amounts of 2× HEPES-buffered saline (2× HBS; 100 μl). The mix was incubated at room temperature for 20 min to allow precipitate formation, while neurons were starved in osmolarity-adjusted NBA (Neurobasal Medium; Thermo Fisher Scientific) at 37°C and 5% CO_2_ for the same time. Precipitates were added to neurons and incubated for 20 min at 37°C and 5% CO_2_. Last, neurons were washed two times with osmolarity-adjusted HBSS (Thermo Fisher Scientific) and transferred back to their conditioned medium, which had been previously saved. In case of HIP1 and HIP1R siRNA knockdown experiments, pHluorin-tagged GluA1 was cotransfected with 50 nM siRNA. Live imaging was conducted at 5 days after transfection (DIV12 to DIV13) in case of cotransfection with siRNA or 7 to 8 days after transfection (DIV14 to DIV16) for all other experiments. Throughout the text, WT hippocampal neurons transfected with an siRNA control or an siRNA against HIP1 or HIP1R are denoted as “CTRL,” “HIP1^KD^,” and “HIP1R^KD^,” respectively.

### Subcellular fractionation from whole brain

All steps were performed at 4°C in the presence of protease inhibitors (cOmplete, EDTA-free protease inhibitor cocktail tablet; Sigma-Aldrich). Mouse brains were homogenized in ice-cold sucrose buffer [320 mM sucrose, 20 mM HEPES, and 5 mM EDTA (pH 7.4)] with 12 strokes at 900 rpm. Samples were then centrifuged at 900*g* for 10 min. The supernatant (S1) was collected; the resulting pellet (P1) contains large cell fragments and nuclei. S1 was then centrifuged at 10,000*g* for 15 min. The supernatant (S2) containing soluble proteins was collected for further analysis, whereas the pellet (P2) containing synaptosomes was carefully resuspended in 4 ml of sucrose buffer and centrifuged at 10,000*g* for 15 min (washing step). The washed crude synaptosomal pellet (P2′) was resuspended in 8 ml of ice-cold ddH_2_O and homogenized by hand with three strokes, and the concentration of HEPES was rapidly adjusted to 4 mM. The lysate was rotated for 30 min to ensure complete lysis and then centrifuged at 25,000*g* for 25 min to separate the synaptosomal membrane fraction (P3) from the synaptosomal cytosol (S3). The P3 pellet was resuspended in 1 ml of sucrose buffer; layered on top of a discontinuous sucrose cushion of 0.8, 1.0, and 1.2 M HEPES-buffered sucrose solution; and centrifuged at 29,100 rpm for 2 hours. Following centrifugation, the synaptic plasma membrane (SPM) fraction at the interphase of 1.0 and 1.2 M sucrose was collected using an 18-gauge needle on a 1-ml syringe. A total of 2.5 volumes of 4 mM HEPES were added to the SPM to adjust the sucrose concentration from 1.2 to 0.32 M. The SPM was then pelleted by centrifugation at 200,000*g* for 30 min. The resulting pellet was resuspended in 300 μl of 50 mM Hepes buffer containing 2 mM EDTA. The SPM was then combined with 2.7 ml of 0.54% Triton X-100 in 50 mM HEPES and 2 mM EDTA followed by centrifugation at 32,000*g* for 20 min. The resulting pellet (PSD fraction) was resuspended in 50 mM HEPES and 2 mM EDTA (pH 7.4). Protein concentration was measured by BCA assay, and equal protein amounts were diluted in 1× Laemmli sample buffer and boiled at 90°C for 10 min. Samples were resolved by SDS–polyacrylamide gel electrophoresis (SDS-PAGE) followed by blotting on nitrocellulose membrane. Membranes were blocked for 1 hour at room temperature using 3% bovine serum albumin (BSA) in 1× PBST [1× phosphate-buffered saline (PBS) and 0.01% Tween 20] and incubated with primary antibodies overnight at 4°C. Membranes were washed three times with 1× PBST and incubated with IRDye 680RD– or IRDye 800CW–conjugated infrared secondary antibodies in 1× PBST for 45 min at room temperature. Afterwards, the membranes were washed three times using 1× PBST and twice with 1× PBS and subsequently analyzed using the Odyssey Fc Imaging System (LI-COR Biosciences) controlled by Image Studio software.

### Synaptosome trypsin cleavage assay

Synaptosomes were prepared as described above and treated with trypsin as previously reported (*26*). All steps were performed at 4°C. Equal amounts of purified synaptosomes (P2) were resuspended in ice-cold sucrose buffer [320 mM sucrose and 5 mM HEPES (pH 8)]. For trypsin cleavage, a trypsin stock solution (0.1 mg/ml) was added to yield a final protein-protease ratio of 100:1. Synaptosomes were incubated for 10 min at 30°C with gentle agitation. The mixture was then centrifuged for 3 min at 8700*g*, and the resulting pellet was directly resuspended in 1× Laemmli sample buffer and boiled at 90°C for 10 min. Samples were resolved by SDS-PAGE and analyzed by immunoblotting using the corresponding primary antibodies. Ratiometric quantification of signal intensities was measured with the supplied Image Studio software package of the Odyssey Fc Imaging System (LI-COR Biosciences).

### Generation of lysates for protein quantification by immunoblotting

All steps were performed at 4°C in the presence of protease inhibitors (cOmplete, protease inhibitor cocktail tablet; Sigma-Aldrich) and phosphatase inhibitors (phosphatase inhibitor cocktail 2 and 3; Sigma-Aldrich). Whole mouse brain or different mouse brain regions were homogenized in ice-cold radioimmunoprecipitation assay (RIPA) buffer (150 mM NaCl, 1% NP-40, 0.5% sodium deoxycholate, 0.1% SDS, and 50 mM Tris-HCl) with 20 strokes at 900 rpm. The lysate was incubated under gentle rotation for 15 min, to ensure complete lysis, before centrifugation at 900*g* for 10 min for removing large cellular debris. Protein concentration was measured by BCA assay. Equal protein amounts were diluted in 1× Laemmli sample buffer, resolved by SDS-PAGE, and analyzed by immunoblotting using the corresponding primary antibodies.

### Time-gated STED imaging of neurons

On DIV14 to DIV16, neurons were fixed using ice-cold methanol fixation solution [for 100 ml: 90 ml of methanol and 10 ml of MES-based solution (100 mM MES, 1 mM EGTA, and 1 mM MgCl_2_; pH 6.9)] for 5 min at −20°C and blocked with 2% BSA in 1× PBS for 30 min. Neurons were then incubated with primary antibodies diluted in 2% BSA in 1× PBS overnight at 4°C, followed by appropriate secondary antibodies diluted 1:400 in 1× PBS for 1 hour at room temperature. Neuronal nuclei were visualized with 4′,6-diamidino-2-phenylindole (DAPI; 1 μg/ml in 1× PBS), and coverslips were mounted using ProLong Gold Antifade (Invitrogen, catalog no. P36930) and stored at 4°C until imaging. STED imaging with time-gated detection was performed on a Leica SP8 TCS STED microscope (Leica Microsystems) equipped with a pulsed white light excitation laser (WLL; ≈80-ps pulse width and 80-MHz repetition rate; NKT Photonics) and two STED lasers for depletion at 592 and 775 nm. The pulsed 775-nm STED laser was triggered by the WLL. Three-channel STED imaging was performed by sequentially exciting ATTO 647N, Alexa Fluor 594, and Alexa Fluor 488 at 646, 598, and 488 nm, respectively. Emission from Alexa Fluor 488 was depleted with the 592-nm laser, whereas the 775-nm STED laser was used to deplete both Alexa Fluor 594 and ATTO 647N. Time-gated detection was set from 0.3 to 6 ns for all dyes. Fluorescence signals were detected sequentially by hybrid detectors at appropriate spectral regions distinct from the STED laser. Images were acquired with an HC PL APO CS2 100×/1.40 numerical aperture (NA) oil objective (Leica Microsystems), a scanning format of 1024 × 1024 pixels, 8-bit sampling, and sixfold zoom, yielding a voxel dimension of 18.9 nm by 18.9 nm. The effective lateral resolution of the TCS SP8 STED microscope was previously determined using 40-nm fluorescent beads (Life Technologies; excitation/emission maxima at 505/515 nm or 660/680 nm) to be 54 nm for the 488-nm channel and 45 nm for the 647-nm channel.

### shRNA design

For CALM shRNA, a 19-nucleotide RNA 5′-GGAAATGGAACCACTAAGA-3′ corresponding to nucleotides 2001 to 2019 of mouse CALM (GenBank accession no: BK001028.1) was designed and used to construct an nuclear localization sequence (NLS)-RFP–containing lentivirus-mediated RNA interference vector targeted to CALM: (f(syn) NLS-RFP CALM shRNA). For HIP1 shRNA, a 19-nucleotide RNA 5′-ACAGAGGTATAGCAAGTTA-3′ corresponding to nucleotides 1461 to 1479 of mouse HIP1 (GenBank accession no: NM_146001.2) was used to construct an NLS-RFP–containing lentivirus-mediated RNA interference vector targeted to HIP1: (f(syn) NLS-RFP HIP1 shRNA). For HIP1R shRNA, a 19-nucleotide RNA 5′-GCTGCTGGCCGCTCAGAGC-3′ corresponding to nucleotides 1842 to 1860 of mouse HIP1R (GenBank accession no: NM_145070.3) was used to construct an NLS-RFP–containing lentivirus-mediated RNA interference vector targeted to HIP1R: (f(syn) NLS-RFP HIP1R shRNA).

### Cell lines

Human embryonic kidney (HEK) 293T cells were obtained from the American Type Culture Collection (catalog no. CRL-3216; RRID:CVCL_0063). Cells were cultured in Dulbecco’s modified Eagle’s medium with glucose (4.5 g/liter; Thermo Fisher Scientific) containing 10% heat-inactivated FBS (Gibco), penicillin (100 U/ml), and streptomycin (100 μg/ml; Gibco) supplemented with 1× nonessential amino acids (Thermo Fisher Scientific) and 1 mM sodium pyruvate (Thermo Fisher Scientific). Cells were routinely tested for mycoplasma contamination.

### Virus production and neuronal transduction

Lentiviral constructs used in this study were all based on the FUGW vector, in which a U6 promoter drives the shRNA transcription, whereas a human synapsin 1 promoter drives the expression of a nuclear-targeted RFP or a cytosolic fluorescent reporter (GFP or mKate) in a neuron-specific manner. The following lentiviral constructs were used in this study: f(syn) eGFP CALM shRNA, f(syn) eGFP scrambled, f(syn) mKate CALM shRNA, f(syn) mKate scrambled (gift from C. Rosenmund), f(syn) NLS-RFP Clathrin shRNA, and f(syn) NLS-RFP scrambled (gift from C. Rosenmund). f(syn) NLS-RFP CALM shRNA, f(syn) NLS-RFP HIP1 shRNA, and f(syn) NLS-RFP HIP1R shRNA were generated for this paper. For lentivirus preparation, HEK293T cells, supplemented with 1× nonessential amino acid solution (Thermo Fisher Scientific) and 1 mM sodium pyruvate (Thermo Fisher Scientific), were transfected using a calcium phosphate–based transfection method with 15 μg of shuttle vector and 10.5 or 4.5 μg of the helper plasmids psPAX2 and pMD2.G, respectively. Twelve hours after transfection, the culture medium was replaced with fresh medium. Supernatants containing viral particles were collected 48 and 72 hours after transfection, filtered to remove cell debris, and concentrated to 30-fold using Amicon tubes (Ultra-15, Ultracel-100k; Merck Millipore). Aliquots were flash-frozen in liquid nitrogen and stored at −80°C until use. For rescue experiments, the following constructs were generated: pAAV-CALM mCherry, pAAV-CALM WT (gift from J. M. Lee), pAAV-CALM Δcargo, pAAV-CALM Δclathrin, pAAV-CALM ΔH0, pAAV-CALM ΔPIP2, and pAAV-CALM Δubiquitin. The preparation of lentiviral and AAV particles (AAV2/9) was performed by the Charité Viral Core Facility (Charité-Universitätsmedizin, Berlin; vcf.charite.de). For both lentiviral and AAV2/9 particles, infection efficiency was determined using WT hippocampal neurons. For all experiments, dissociated neurons were infected at DIV2. In all cases, an infection rate of over 95% was achieved at DIV14 to DIV16. For detection of expression levels by Western blot, neurons were lysed and harvested at DIV14 to DIV16 using RIPA buffer supplemented with protease inhibitors (cOmplete protease inhibitor cocktail tablet; Sigma-Aldrich) and phosphatase inhibitors (phosphatase inhibitor cocktail 2 and 3; Sigma-Aldrich). Proteins were analyzed by SDS-PAGE and immunoblotting using corresponding primary antibodies. Ratiometric quantification of signal intensities was measured with the supplied Image Studio software package of the Odyssey Fc Imaging System (LI-COR Biosciences). Throughout the text and figures, WT hippocampal neurons expressing a scrambled shRNA, an anti-CALM shRNA, an anti-CHC shRNA, an anti-HIP1 shRNA, or an anti-HIP1R shRNA are denoted as “CTRL,” “CALM^KD^,” “CHC^KD^,” “HIP1^KD^,” and “HIP1R^KD^,” respectively.

### Transferrin uptake

For transferrin uptake, cultured hippocampal neurons at DIV14 to DIV16 were starved for 1 hour in serum-free and osmolarity-adjusted NBA medium at 37°C and 5% CO_2_. Subsequently, neurons were treated with transferrin (25 μg/ml) coupled to Alexa Fluor 647 (Thermo Fisher Scientific, catalog no. A-21236; RRID:AB_2535805) in osmolarity-adjusted NBA medium for 20 min at 37°C and 5% CO_2_. To remove unbound Tf-647, neurons were washed twice with cold 1× PBS supplemented with 10 mM MgCl_2_, once with cold 0.1 M acetic acid supplemented with 0.2 M NaCl to quench surface-bound Tf-647, and finally twice with cold 1× PBS supplemented with 10 mM MgCl_2_ before 15 min fixation at room temperature with 4% (w/v) paraformaldehyde (PFA) and 4% sucrose in 1× PBS. Neuronal nuclei were visualized with DAPI (1 μg/ml in 1× PBS), and coverslips were mounted using Immu-Mount (Thermo Fisher Scientific). Transferrin uptake was analyzed using a Zeiss LSM 710 laser scanning confocal microscope with a 63× oil objective. All acquisition settings were set equally for all groups within each experiment. Confocal stacks were analyzed using ImageJ [National Institutes of Health (NIH)]. For quantitative analysis of fluorescence intensities, the area of the neurons (including the cell body and dendrites) was manually selected using the ImageJ selection tools (ROI Manager), and mean intensity was quantified within the region of interest (ROI).

### GluA1/A2 antibody feeding assay

Endocytosis of AMPARs from the cell surface to intracellular compartments was visualized and quantified by an “antibody feeding” assay using cultured hippocampal neurons at DIV14 to DIV16. Neurons were gently washed once using osmolarity-adjusted HBS [25 mM HEPES, 140 mM NaCl, 5 mM KCl, 1.8 mM CaCl_2_, 0.8 mM MgCl_2_, and 10 mM glucose (pH 7.4)] before live labeling surface-localized GluA1 or GluA2 with specific monoclonal antibodies (20 μg/ml) against their extracellular N-terminal regions for 15 min at 37°C. Neurons were fixed for 5 min at room temperature with 4% (w/v) PFA and 4% sucrose in PBS (1×) without permeabilization and stained with Alexa Fluor 568 secondary antibody for 45 min at room temperature to visualize surface-localized receptors (surface pool). Neurons were then permeabilized for 5 min using 0.1% Triton X-100 and stained with Alexa Fluor 647–conjugated secondary antibodies for 45 min at room temperature to visualize internalized receptors (internal pool). Samples were visualized using a Zeiss LSM 710 laser scanning confocal microscope using a 63× oil objective. All acquisition settings were set equally for all groups within each experiment. Confocal stacks were analyzed using ImageJ (NIH). Surface and internal levels were individually quantified for each cell body and dendrites, and a ratio between the mean intensities of internal to surface was generated for WT and KO conditions.

### Chemical LTD induction and pSer^845^-GluA1 dephosphorylation assay

For antibody-feeding experiments, live hippocampal neurons (DIV15 to DIV17) expressing either HA-SEP-GluA1 or myc-SEP-GluA2 were treated with 1 μM TTX in neuronal medium for 30 min. Neurons were surface-labeled with anti-GFP antibodies for 15 min at 37°C in ACSF-LTD [25 mM HEPES, 119 mM NaCl, 2.5 mM KCl, 2 mM CaCl_2_, 0.8 mM MgCl_2_, and 10 mM glucose (pH 7.4)] and washed once in ACSF-LTD before chemical LTD was induced using ACSF-LTD containing 50 μM NMDA, 20 μM glycine, and 1 μM TTX. After 5 min of stimulation at 37°C, neurons were replaced in regular ACSF-LTD containing 1 μM TTX for additional 5 min. Neurons were fixed for 5 min with 4% PFA + 4% sucrose and stained with anti-mouse Alexa Fluor 568–conjugated secondary antibodies. Cells were permeabilized and stained with anti-mouse Alexa Fluor 647–conjugated secondary antibodies. Images were acquired on a Zeiss LSM 710 laser scanning confocal microscope with a 63× oil objective and analyzed using ImageJ software. The internalization index was calculated by dividing the value corresponding to internalized fluorescence by the value corresponding to surface fluorescence.

Chemical LTD was also used for monitoring GluA1 dephosphorylation (pSer^845^). Upon LTD induction, hippocampal neurons were subjected to cell lysis. Protein concentration was determined by BCA assay. Proteins were resolved by SDS-PAGE and analyzed by immunoblotting using corresponding primary antibodies.

### pHluorin imaging of living hippocampal neurons

Hippocampal neurons were imaged at room temperature in *N*-tris(hydroxy-methyl)-methyl-2-aminoethane-sulphonic acid (TES)–based physiological imaging buffer [170 mM NaCl, 3.5 mM KCl, 0.4 mM KH_2_PO_4_, 20 mM TES, 5 mM NaHCO_3_, 5 mM glucose, 1.2 mM Na_2_SO_4_, 1.2 mM MgCl_2_, and 1.3 mM CaCl_2_ (pH 7.4)] by epifluorescence microscopy [Nikon Eclipse Ti, eGFP filter set F36-526, and a scientific complementary metal-oxide semiconductor (sCMOS) camera (Neo, Andor) equipped with a 40× oil-immersion objective] and operated by open-source ImageJ-based MicroManager 4.11 software. In initial optimization experiments, the imaging parameters were established to ensure minimal photobleaching of fluorescence. For all pHluorin-tagged proteins, fluorescence was stable during the imaging experiments (time, >5 min). Images were acquired every 2 s with 100-ms excitation at 488 nm. To measure the steady-state surface to total ratios, the surface-localized pHluorins (*F*_S_) were first quenched by replacing the extracellular TES solution by MES (pH 5.5). To measure the total fluorescence of overexpressed pHluorins (*F*_T_), 50 mM NaCl were replaced by NH_4_Cl. Regions for analysis were selected manually, and quantitative analysis was performed using ImageJ (NIH). The surface-to-total ratio was calculated as (baseline − *F*_S_)/(*F*_T_ − *F*_S_). Our analysis was restricted to dendritic areas for pHluorin-tagged GluA1, GluA2, and TrfR and to axonal regions for synaptophysin.

### Fluorescence imaging of endocytosis events

Dissociated hippocampal neurons from embryonic day 18 rat embryos were plated on 18-mm poly-d-lysine–coated glass coverslips at a density of 50,000 cells/ml in MEM containing 10% horse serum (Invitrogen) for 3 hours and then cultured in Neurobasal medium supplemented with 2 mM glutamine and 10% B27 (Gibco) on a feeder layer of glial cells at 37°C in 5% CO_2_ for 13 to 21 days. CALM-mCherry was expressed under the human synapsin promoter. Neurons (15 DIV, transfected at DIV8 with CALM-mCherry and SEP-GluA1) were perfused with HBS solution at 37°C. HBS contained 120 mM NaCl, 2 mM KCl, 2 mM MgCl_2_, 2 mM CaCl_2_, 5 mM d-glucose, and 10 mM HEPES and was adjusted to pH 7.4 and 260 to 270 mOsm. For the ppH assay, MES-buffered saline solution (MBS) was prepared similarly by replacing HEPES with MES and adjusting the pH to 5.5. All salts were from Sigma-Aldrich. HBS and MBS were perfused locally around the recorded cell using a two-way borosilicate glass pipette. Imaging was performed with an Olympus IX71 inverted microscope equipped for total internal reflection fluorescence (TIRF) microscopy with a 150×, 1.45 NA objective (UAPON150XOTIRF), a laser source (Cobolt Laser 06-DPL 473 nm, 100 mW), and an ILas2 illuminator (Gataca Systems) with a penetration depth set to 100 nm. Emitted fluorescence was filtered with a dichroic mirror (R405/488/561/635) and an emission filter (ET525/50m, Chroma Technology) and recorded by an electron-multiplying charge-coupled device (EMCCD) camera (QuantEM 512C, Princeton Instruments). Movies were acquired for 5 min at 0.5 Hz. Semiautomatic detection of endocytic events and their analysis were conducted using custom-made MATLAB scripts as described in (*76*). In short, a sudden, punctate fluorescence increase appearing in pH 5.5 images was detected as being an endocytic event if (i) it was visible for more than three frames (i.e., 8 s) and (ii) it appeared at the same location as a preexisting fluorescence cluster detectable in pH 7.4 images. For fluorescence quantification, each value was calculated as the mean intensity in a 2-pixel radius circle centered on the detection to which the local background intensity is subtracted (the local background is taken as the 20th to 80th percentile of fluorescence in an annulus of 5- to 2-pixel outer and inner radii centered on the detection). Ninety-five percent intervals for significant recruitment were determined by measuring the fluorescence of 200 random shifts within the cell mask. Colocalization between markers was quantified with the Coloc2 plugin of ImageJ to calculate Pearson’s *R* coefficient on selected portions of dendrites as ROIs with automatic threshold determination. For each ROI, we performed 20 randomizations to calculate corresponding *r* coefficients, which were systematically below the actual measure, confirming the specific colocalization.

### Surface biotinylation of cortico-hippocampal cultures

To biotinylate all surface proteins, after washing once with ice-cold PBS^2+^ [137 mM NaCl, 2.7 mM KCl, 10 mM Na_2_HPO_4_, 1.8 mM KH_2_PO_4_, 1 mM CaCl_2_, and 0.5 mM MgCl_2_ (pH 7.4)], cortico-hippocampal neurons at DIV12 to DIV14 in six-well plates were incubated in the dark with the non–membrane-permeant, cleavable biotin derivative, sulfo-NHS-SS-biotin (0.5 mg/ml; EZ-Link, Pierce Thermo Fisher Scientific), for 20 min under gentle shaking at 4°C. After biotinylation, neurons were washed twice with ice-cold quenching solution (50 mM glycine in PBS^2+^) for 5 min to remove excess biotin. Then, neurons were rinsed once with ice-cold PBS^2+^ and immediately solubilized in RIPA buffer supplemented with protease inhibitors (cOmplete protease inhibitor cocktail tablet; Sigma-Aldrich) and phosphatase inhibitors (phosphatase inhibitor cocktail 2 and 3; Sigma-Aldrich). Lysates were cleared by centrifugation at 13,000*g* for 10 min at 4°C; supernatants were collected, and protein concentration was determined by BCA assay. A portion of the supernatant was collected and used as input. To precipitate the biotinylated proteins, equal amounts of total protein were incubated with streptavidin agarose beads (Thermo Fisher Scientific) at 4°C with rotation. After 2 hours, beads were extensively washed with RIPA buffer, and surface biotinylated proteins were eluted by boiling for 10 min in 1× Laemmli sample buffer, resolved by SDS-PAGE, and immunoblotted using appropriate antibodies. Equal amounts of total cell lysates were analyzed for total protein levels. Ratiometric quantification of signal intensities was measured with the supplied Image Studio software package of the Odyssey Fc Imaging System (LI-COR Biosciences).

### Photobleaching (FRAP) experiments

Hippocampal neurons were cotransfected at DIV7 to DIV8 with SEP-GluA1 and spinophilin-mCherry to identify both synaptic and extrasynaptic regions. At DIV14 to DIV16, neurons were imaged in physiological imaging buffer [170 mM NaCl, 3.5 mM KCl, 0.4 mM KH_2_PO_4_, 20 mM TES, 5 mM NaHCO_3_, 5 mM glucose, 1.2 mM Na_2_SO_4_, 1.2 mM MgCl_2_, and 1.3 mM CaCl_2_ (pH 7.4)] in a heated chamber at 37°C. Images were acquired using a Zeiss LSM 710 confocal laser scanning microscope supported by a ZEN 2010 software. For FRAP, the 488-nm line of the argon laser and the 561-nm line of the diode-pumped solid-state laser (DPSSL) were used in combination with a Plan Apochromat 63×/1.40 oil differential interference contrast objective. Time series were collected as repetitively scanned single confocal slices. After 20 s of baseline recording, the 488-nm laser power was increased to 100%, and a predefined circular ROI was bleached by a single laser scan. A total time of 300 frames after bleaching were acquired with 2-s intervals. Analysis was conducted using ImageJ (NIH). Photobleaching due to image acquisition was corrected by normalization to nonphotobleached synaptic or extrasynaptic regions, distant to the bleached synaptic or extrasynaptic region, respectively. Recovery curves were normalized to the fluorescence measured before the bleach, and residual fluorescence right after the bleach was set to zero.

### Immunohistochemical analysis

Mice were anesthetized by intraperitoneal application of ketamine/xylazine and transcardially perfused with 16 ml of 1× PBS (room temperature), followed by ca. 25 ml of 4% (w/v) PFA (Merck) in phosphate buffer [PB; 0.125 mM Na_2_HPO_4_/NaH_2_PO_4_ (pH 7.4); room temperature] at a speed of 8 ml/min. Brains were carefully taken out of the skull, postfixed overnight in the same fixative, and placed in dimethyl sulfoxide (DMSO) solution [a mixture of 20% (v/v) glycerol and 2% (v/v) DMSO (VWR International) in 0.4 M PB] for 24 hours for cryoprotection. Frozen horizontal, coronal, or sagittal sections were collected in six series in DMSO solution. For immunostaining, corresponding hippocampal sections from WT and KO littermates were processed simultaneously. To detect surface-stranded GluA1 or GluA2 in slices, sections were washed for 3 hours in PB (exchanging solution every 20 min), blocked in PB containing 5% (v/v) normal goat serum, and incubated overnight at 4°C with a primary antibody directed against the N-terminal region of GluA1 or GluA2. Unbound antibody was removed by washing in PB for 2 hours (exchanging the solution every 15 min), and bound antibodies were decorated with an Alexa Fluor 488–conjugated secondary antibody for 1.5 hours in PB to detect the surface pool. Subsequently, sections were washed in PB for 45 min and postfixed with 2% (v/v) PFA, followed by a washing step (3× 15 min in PB) and permeabilization with PB containing 0.3% Triton X-100. To stain the total pool of glutamate receptors, the first part of the protocol was repeated under permeabilizing conditions, and an Alexa Fluor 568–conjugated secondary antibody was used. Last, nuclei were stained with DAPI (1 μg/ml in PB), and sections were mounted on gelatin-coated glass slides. Images of brain slices were acquired in a blind manner using a Zeiss LSM 710 confocal laser scanning microscope with a 40× oil objective. All acquisition settings were set equally for sections of all groups within each immunostaining. Fluorescence levels were quantified using ImageJ (NIH), measuring the mean intensity in defined ROIs of the cortex and CA1/CA3 regions in the hippocampus. To quantify the surface levels of GluA1 and GluA2, a ratio between the mean intensities of surface to total within each ROI was generated for WT and KO conditions.

### Organotypic slice cultures and rectification index measurements

Organotypic hippocampal cultures were prepared from p6 to p9 WT C57BL/6JCrl mice. Slices (350 μm thick) were cultured according to the interface method in a MEM-based culture medium with the addition of 5% horse serum, 1× B27, 25 mM HEPES, 3 mM l-glutamine, 2.8 mM CaCl_2_, 1.8 mM MgSO_4_, 0.25 mM ascorbic acid, and d-glucose (6.5 g/liter). The medium was replaced every 4 days. Cultures were grown in an incubator with 5% CO_2_ at 34°C. Lentiviral shRNA constructs [f(syn) NLS-RFP scramble or f(syn) NLS-RFP CALM shRNA] were added at DIV3 to DIV4. pAAV-CALM mCherry was added 2 days before recordings. Somatic whole-cell recordings were performed on visually identified CA1 principal neurons at 17 to 23 DIV with borosilicate glass microelectrodes (3 to 8 megohms) filled with intracellular solution containing 135 mM K·CH_3_SO_3_, 4 mM NaCl, 2 mM MgCl_2_, 2 mM Na_2_ATP, 0.3 mM Na_2_GTP, 0.06 mM EGTA, 0.01 mM CaCl_2_, and 10 mM HEPES, adjusted to 300/310 mOsm/liter and pH 7.2. Slices were superfused with a recirculating ACSF (5 ml/min at 24°C) containing the following: 145 mM NaCl, 2.5 mM KCl, 2 mM CaCl_2_, 1 mM MgCl_2_, 10 mM HEPES, and 10 mM glucose, adjusted to 305/315 mOsm/liter and pH 7.3 with NaOH. The extracellular solution was supplemented with the following: MNI-caged-l-glutamate (0.5 mM; Hello Bio, HB0423) and AP-5 (20 μM; Hello Bio), SR-99531 (10 μM; Hello Bio), UBP-310 (10 μM; Hello Bio), and TTX (1 μM; Hello Bio) to block NMDARs, GABA receptors, kainate receptors, and sodium channels, respectively. To perform rectification index experiments on visualized subareas of the dendritic trees of principal neurons, we added the fluorophore Alexa Fluor 594 (20 μM; Thermo Fisher Scientific) to the intracellular solution. Once the whole-cell modality was reached, the dye was allowed to diffuse for several minutes into the neuronal cytoplasm. Then, ultraviolet light pulses with a diameter of roughly 1 μm (duration, 5 ms) were delivered at the same dendritic location at −60-mV and +40-mV holding potentials (not corrected for the liquid junction potential, which was estimated to be −6.6 mV) to estimate the rectification index. Recordings with series resistance changes >20% were discarded. Simultaneous passage of red emission (600 nm) and 405-nm light for uncaging was achieved by using a 405/488/594-nm Laser Triple Band filter set (TRF 69902; Chroma) mounted in a Zeiss TIRF cube.

### Immunoprecipitations

For immunoprecipitation experiments, all steps were performed at 4°C in the presence of protease inhibitors (cOmplete, EDTA-free protease inhibitor cocktail tablet; Sigma-Aldrich) and phosphatase inhibitors (phosphatase inhibitor cocktail 2 and 3; Sigma-Aldrich). P3 (synaptosomal membrane fraction) was prepared as described above and resuspended in immunoprecipitation lysis buffer [50 mM tris-HCl, 150 mM NaCl, and 1% DMM (*n*-dodecyl β-d-maltoside; Sigma-Aldrich)]. Protein concentration was measured by BCA. P3 lysate (4 mg) was incubated with 3 μg of antibody or with an equivalent amount of IgG control for 1 hour on a rotating wheel before the addition of 30 μl of Pierce Protein A/G Magnetic Beads (Thermo Fisher Scientific) for an additional 3 hours. Following incubation, samples were washed four times with washing buffer [50 mM tris-HCl, 120 mM NaCl, and 0.5% DMM (Sigma-Aldrich)] and proteins were eluted with 1× Laemmli sample buffer, resolved by SDS-PAGE, and analyzed by immunoblotting.

### Pull-down assays

For pull-down assays, all steps were performed at 4°C. GST-fusion proteins were expressed in *E. coli* (BL21) at 16°C for 16 to 18 hours and coupled to Glutathione Sepharose beads (Novagen) according to the manufacturer’s instructions. Whole mouse brain extract was prepared using pull-down lysis buffer (10 mM HEPES and 1% Triton X-100) in the presence of protease inhibitors (cOmplete, protease inhibitor cocktail tablet; Sigma-Aldrich) and phosphatase inhibitors (phosphatase inhibitor cocktail 2 and 3; Sigma-Aldrich). Protein concentration was measured by BCA. GST (30 μg) or GST-fusion protein (30 μg) was incubated with 4 mg of whole mouse brain extract overnight under constant rotation at 4°C. Samples were washed three times using pull-down lysis buffer, boiled with 1× Laemmli sample buffer, resolved by SDS-PAGE, and analyzed by immunoblotting using corresponding antibodies.

### In vitro binding assays

All GST-fusion proteins and His_6_-tagged proteins were expressed in *E. coli* (BL21) at 16°C for 16 to 18 hours and coupled to Glutathione Sepharose beads (Novagen) or HIS-Select Nickel Affinity Gel (Sigma-Aldrich), respectively, according to the manufacturer’s instructions. Equal amounts (5 μg) of recombinant purified GST or GST-fusion proteins and His-fusion proteins were incubated in 300 μl of binding buffer [20 mM HEPES (pH 7.4), 100 mM NaCl, 2 mM MgCl_2_, and 0.1% saponin] for 1 hour at 4°C on a rotating wheel. Samples were washed three times in binding buffer, and proteins were eluted in 1× Laemmli sample buffer. Proteins (1 μg) were resolved by SDS-PAGE and analyzed by immunoblotting using GST- and His-tag specific antibodies.

### Statistical analysis

Detailed statistical information is provided in Table 1. Data are depicted as means ± SEM and represent values from several independent experiments (*n*, neuronal cultures or slices; *N*, animal pairs) as indicated in the figure legends. Statistical evaluation of differences between groups was based on at least three independent experiments. For comparisons between two experimental groups, statistical significance was evaluated using two-tailed unpaired Student’s *t* test. When more than two groups were compared, data were analyzed by one-way ANOVA followed by a Dunnett’s post hoc test or in combination with the Holm-Sidak method. Where data had to be normalized before analyses, one-sample *t* tests were used for comparisons with control group values that had been set to 100. To compare observed distributions with expected distributions, a two-tailed binomial test (Wilson/Brown) was used. Significance levels are indicated as *P < 0.05, **P < 0.01, ***P < 0.005, and ****P < 0.0001. Differences that are not significant are indicated as ns. Statistical data evaluation was performed using GraphPad Prism 9.3.1 (471). All figures were assembled using Affinity Designer. For Fig. 3 (A to C) and fig. S3A, data were evaluated in a blinded manner.

## Notes

View/request a protocol for this paper from *Bio-protocol*.
