# Supplementary material for: Selective endocytosis of Ca2+-permeable AMPARs by the Alzheimer’s disease risk factor CALM bidirectionally controls synaptic plasticity
Source: Sci Adv. 2022 May 25;8(21):eabl5032. doi: 10.1126/sciadv.abl5032 (PMC9132451; doi:10.1126/sciadv.abl5032)
Supplement: Supplementary file 1 — Figs. S1 to S7 [file sciadv.abl5032_sm.pdf]

Science Advances
AAAS

# Supplementary Materials for

## Selective endocytosis of $Ca^{2+}$-permeable AMPARs by the Alzheimer's disease risk factor CALM bidirectionally controls synaptic plasticity

Domenico Azarnia Tehran *et al.*

Corresponding author: Domenico Azarnia Tehran, azarnia@fmp-berlin.de; Volker Haucke, haucke@fmp-berlin.de; Tanja Maritzen, maritzen@bio.uni-kl.de

*Sci. Adv.* **8**, eabl5032 (2022)
DOI: 10.1126/sciadv.abl5032

**This PDF file includes:**

Figs. S1 to S7

**Figure S1**

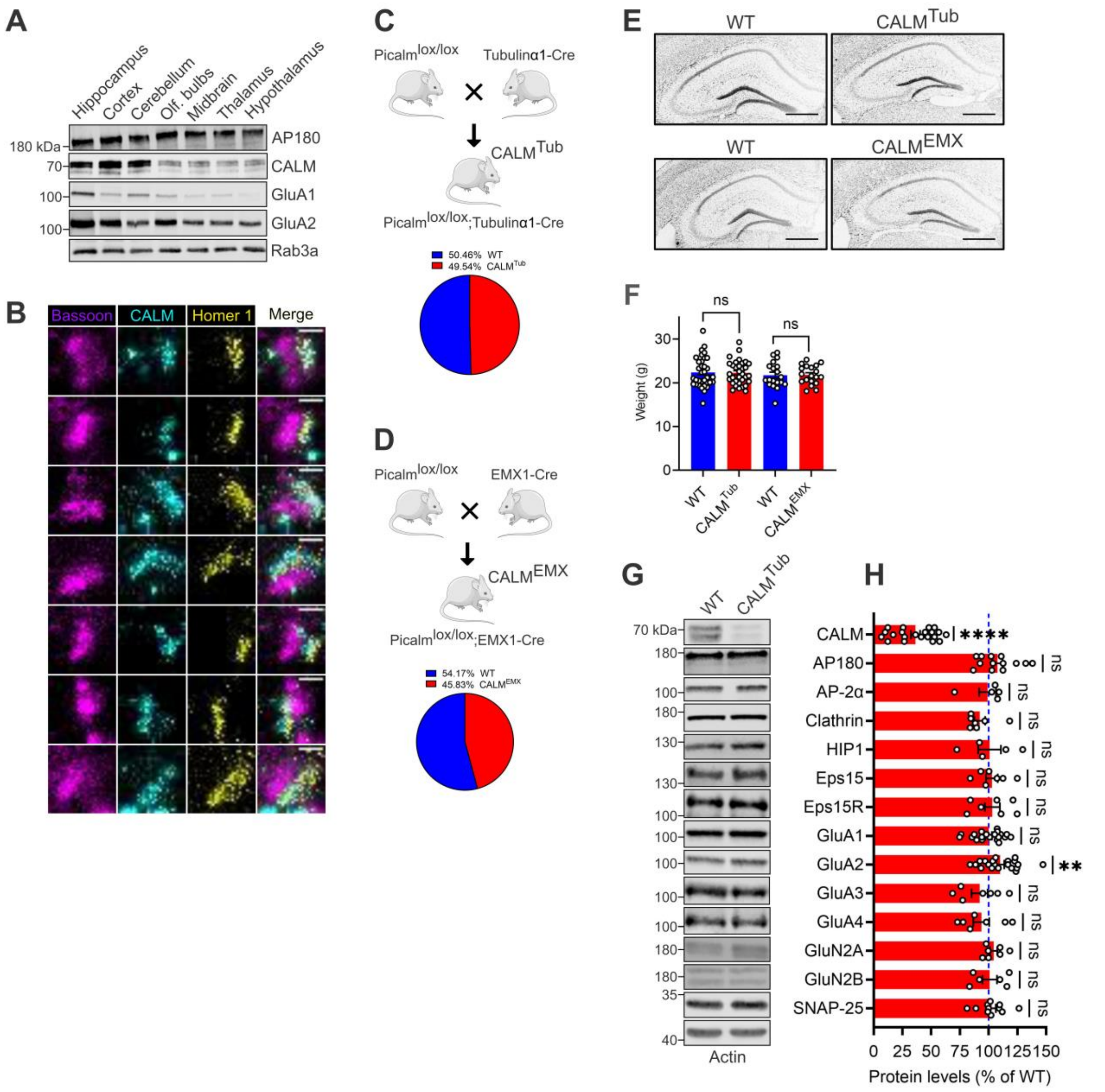


**Fig. S1. Generation and basic characterization of neuron-specific CALM KO mouse lines.** (**A**) CALM is expressed throughout the murine brain with highest levels in hippocampus, cortex and cerebellum. Lysates of different brain regions from adult mice were probed by immunoblotting with the indicated antibodies. (**B**) Representative images of WT hippocampal neurons immunolabeled with antibodies against the presynaptic marker Bassoon, the postsynaptic marker Homer 1 as well as CALM and imaged by gSTED (scale bar, 500 nm). (**C** and **D**) Illustration of breeding schemes and analysis of genotype distribution of 216 offspring from CALM lox/lox x CALM lox/lox Tubulin α1-Cre$^{+/-}$ breedings (CALM$^{Tub}$) (C) and of 192

offspring from CALM lox/lox x CALM lox/lox EMX1-Cre$^{+/-}$ crossings (CALM$^{EMX}$) (D) showing the expected ~1:1 Mendelian distribution (binomial test). (**E**) No alterations in gross brain anatomy of CALM KO mouse models. Representative confocal images of PFA-fixed and DAPI-stained brain sections of CALM KO$^{Tub}$ or CALM KO$^{EMX}$ mice and their respective WT littermates (scale bar: 500 µm). (**F**) Unaltered body weights of adult CALM KO$^{Tub}$ or CALM KO$^{EMX}$ mice and their WT littermates. Data represent mean±SEM (N=at least 19; two-tailed unpaired t-test). (**G** and **H**) Levels of synaptic and endocytic proteins in the brain are mostly unaltered in absence of neuronal CALM. Total brain lysates from adult WT and CALM KO$^{Tub}$ mice probed by immunoblotting with the indicated antibodies. Representative immunoblot (G) and quantification (H) of protein levels in CALM KO$^{Tub}$ brains normalized to WT. Data represent mean±SEM (N=at least 5; one sample t-test).

**Figure S2**

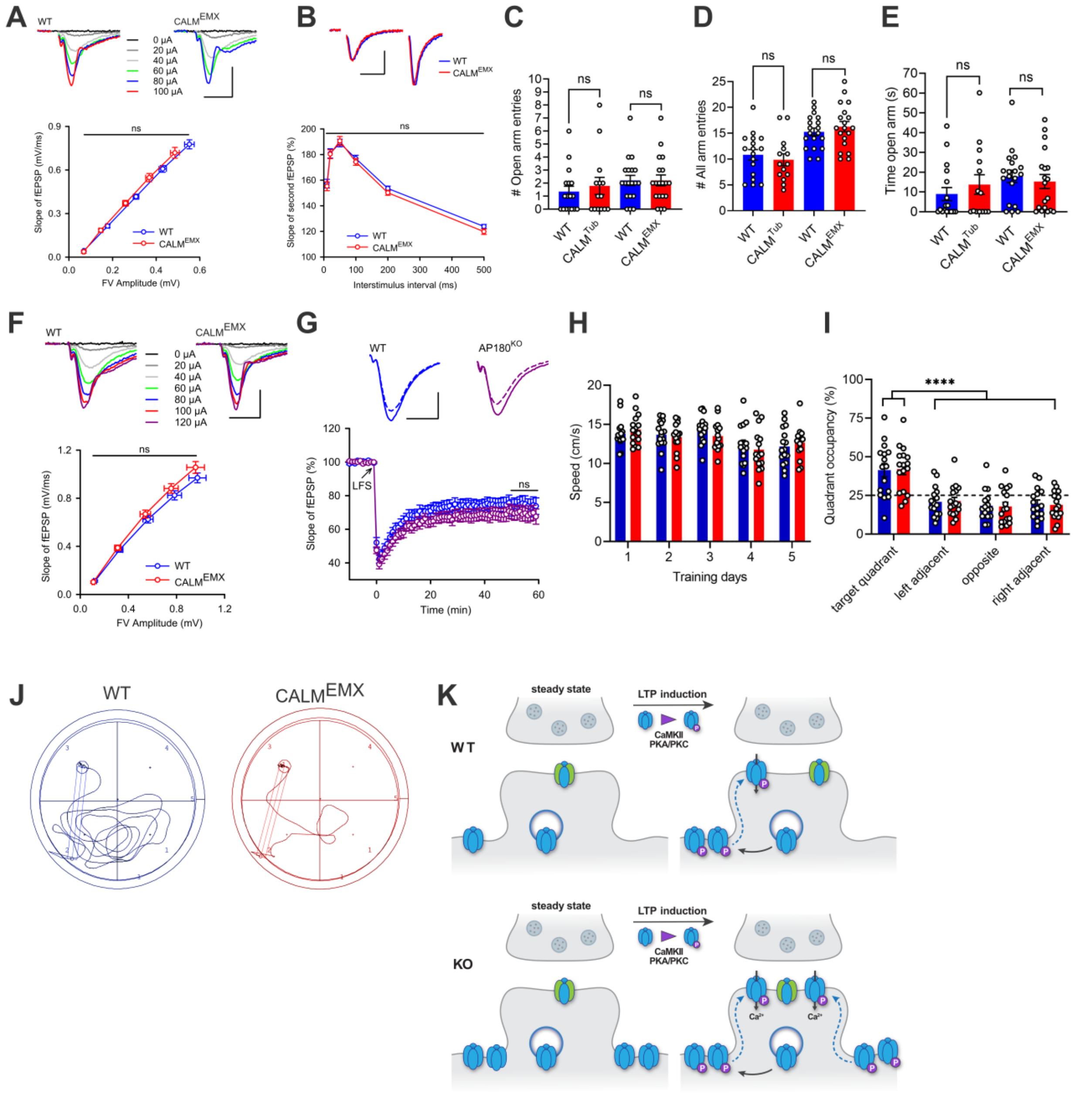


**Fig. S2. Electrophysiological and behavioral characterization of CALM KO$^{EMX}$ mice.**

(**A**) Unaltered basal synaptic transmission in two-months-old CALM$^{EMX}$ mice. The input-output relationship of fEPSP slopes *vs* fiber volley amplitudes over a range of stimulation intensities reveal no significant difference between WT and CALM$^{EMX}$ mice. Inserts represent exemplary traces recorded in WT and CALM$^{EMX}$ slices and evoked by increasing stimulation intensities until maximal fEPSPs were obtained (scale bar: 1 mV and 10 ms) (WT: N=13 animals, n=25 slices; CALM$^{EMX}$: N=12 animals, n=23 slices; two-way repeated measures ANOVA). (**B**)

Unaltered paired-pulse facilitation (PPF) in two-months-old CALM$^{EMX}$ mice. The facilitation of second responses over a range of interstimulus intervals (10–500 ms), measured as ratios of fEPSP slopes, reveal no significant difference between WT and CALM$^{EMX}$ mice. Inserts represent exemplary traces of fEPSPs at 50 ms interstimulus interval from WT and CALM$^{EMX}$ slices (scale bar: 0.5 mV and 20 ms) (WT: N=13 animals, n=25 slices; CALM$^{EMX}$: N=12 animals, n=23 slices; two-way repeated measures ANOVA). (**C** to **E**) CALM KO$^{Tub}$ mice do not display altered anxiety-related behaviour. Mice of the indicated genotypes were tested for 5 min on an Elevated Plus Maze, and the number of open arm visits (C), all arm entries (D) and the time spent on the open arms (E) were scored (N=at least 14; two-tailed unpaired t-test). (**F**) Unaltered basal synaptic transmission in two-weeks-old CALM$^{EMX}$ mice. The input-output relationship of fEPSP slopes *vs* fiber volley amplitudes over a range of stimulation intensities reveal no significant difference between WT and CALM$^{EMX}$ mice. Inserts represent exemplary traces recorded in WT and CALM$^{EMX}$ slices evoked by increasing stimulation intensities until maximal fEPSPs were obtained (scale bar: 1 mV and 10 ms) (WT: N=11 animals, n=22 slices; CALM$^{EMX}$: N=11 animals, n=22 slices; two-way repeated measures ANOVA). (**G**) Long-term depression (LTD) is not altered in two-months-old AP180$^{KO}$ mice. The graph illustrates the time course of LTD induced by low frequency stimulation (LFS), which is not affected in AP180$^{KO}$ mice. Each value is an average of 3 consecutive time points recorded every 20 s and the mean slopes of the fEPSPs recorded 0-10 min before LFS are taken as 100%. The representative fEPSPs recorded 0-10 min before (solid line) and 50-60 min after LFS (dashed line) are shown above (scale bar: 0.5 mV and 10 ms) (WT: N=6 animals, n=10 slices; AP180$^{KO}$: N=6 animals, n=11 slices; two-tailed unpaired t-test). (**H**) Swimming speed over 5 days of training during the Morris water maze (WT: N=16 animals; CALM$^{EMX}$: N=18 animals) (**I**) Quadrant occupancy during probe trial on day 5 (WT: N=16 animals; CALM$^{EMX}$: N=17 animals; one-way ANOVA followed by Dunnett´s post hoc test). (**J**) Representative swim tracks of WT and CALM KO$^{EMX}$ mice on day 2 of training. (**K**) Illustration of mechanism underlying differences in LTP between WT and CALM KO mice.

**Figure S3**

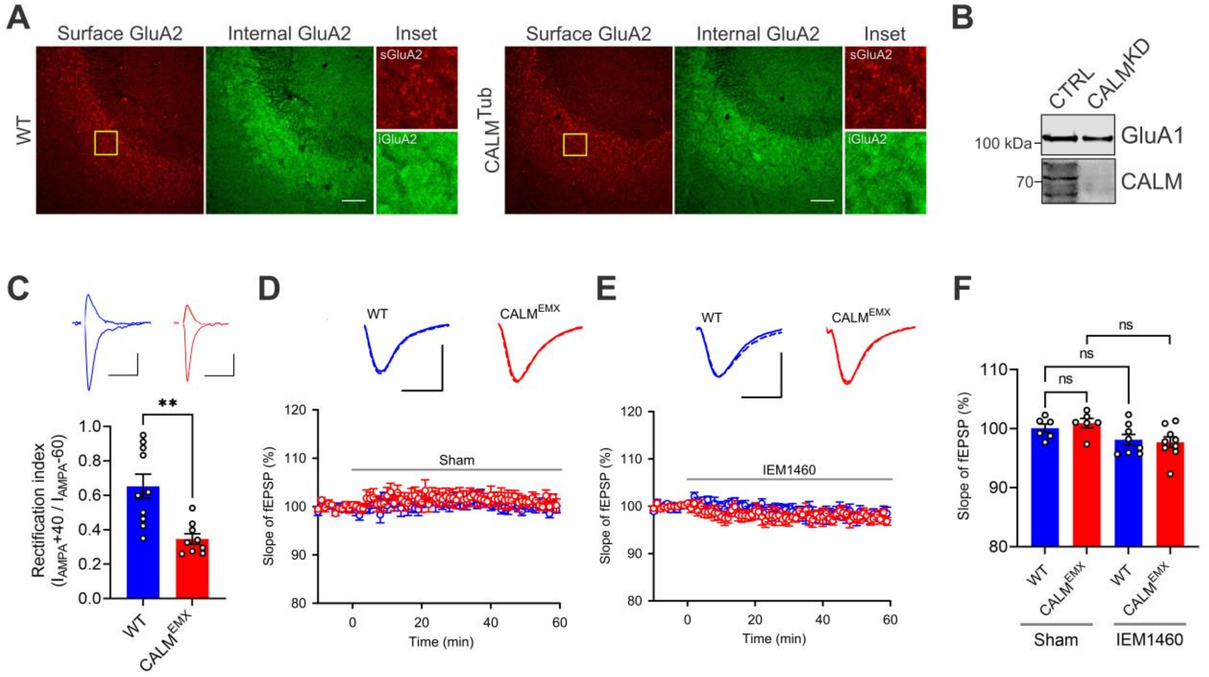


**Fig. S3. CP-AMPARs are largely dispensable for basal neurotransmission.**

(**A**) Representative confocal images of perfused sagittal brain sections of WT and CALM KO$^{Tub}$ mice immunostained for GluA2 either in absence or in presence of detergent to label the surface (red) or the internal (green) pool (scale bar: 50 µm). (**B**) Efficient depletion of CALM by shRNA-based knockdown (CALM$^{KD}$) in organotypic slices cultures. Lysates obtained from WT organotypic cultures transduced with lentivirus expressing either an inactive scrambled shRNA (CTRL) or an anti-CALM shRNA (CALM$^{KD}$) were analyzed by immunoblotting with antibodies against CALM and GluA1. (**C**) Representative traces of whole-cell recordings from CA1 principal neurons upon electrical stimulation of the Schaffer collateral in WT and CALM KO$^{EMX}$ acute slices (scale bars: 50 pA and 100 ms). Quantification of the AMPAR-dependent rectification index shows a significant reduction in KO slices (n=10 neurons from N=3 WT animals and n=9 neurons from N=3 CALM KO$^{EMX}$ animals; two-tailed unpaired t-test). (**D** to **F**) Basal synaptic transmission is unaffected by loss of CALM. AMPAR-mediated responses were recorded in WT and CALM KO$^{EMX}$ slices under basal conditions with sham control (D) or in the presence of the CP-AMPAR inhibitor IEM 1460 (E). Representative fEPSPs recorded 0-10 min before (solid line), and 50-60 min after application of treatment (dashed line) are shown above (scale bars: 1 mV and 10 ms). (F) Values of fEPSP slope quantified as % change of the responses during the last 10 min (Sham: WT N=6 animals, n=6 slices; CALM KO$^{EMX}$ N=6

animals, n=6 slices; IEM 1460: WT N=6 animals, n=8 slices; CALM KO$^{EMX}$ N=6 animals, n=9 slices; one-way ANOVA with Holm-Sidak).

# Figure S4

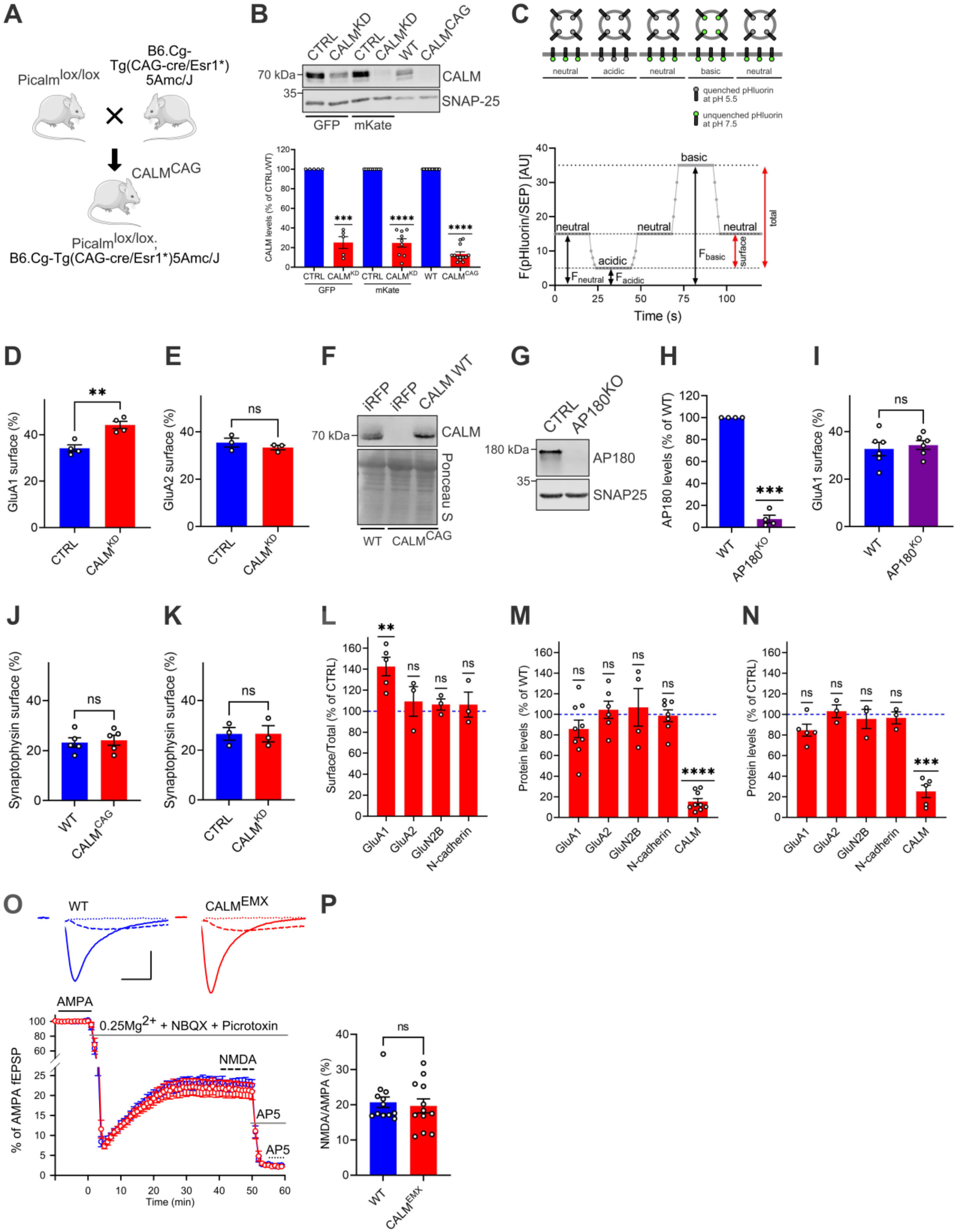

A
Picalm$^{lox/lox}$
B6.Cg-Tg(CAG-cre/Esr1*)5Amc/J
CALM$^{CAG}$
Picalm$^{lox/lox}$; B6.Cg-Tg(CAG-cre/Esr1*)5Amc/J
B
CTRL CALMKD CTRL CALMKD WT CALMCAG
70 kDa
35
CALM
SNAP-25
GFP mKate
CALM levels (% of CTRL/WT)
C
neutral acidic neutral basic neutral
quenched pHluorin at pH 5.5
unquenched pHluorin at pH 7.5
F(pHluorin/SEP) [AU]
Time (s)
total
surface
D
GluA1 surface (%)
E
GluA2 surface (%)
F
iRFP iRFP CALM WT
CALM
Ponceau S
WT CALM$^{CAG}$
G
CTRL AP180KO
180 kDa
AP180
SNAP25
H
AP180 levels (% of WT)
I
GluA1 surface (%)
J
Synaptophysin surface (%)
K
Synaptophysin surface (%)
L
Surface/Total (% of CTRL)
M
Protein levels (% of WT)
N
Protein levels (% of CTRL)
O
WT
CALM$^{EMX}$
AMPA
0.25Mg$^{2+}$ + NBQX + Picrotoxin
NMDA
AP5
% of AMPA fEPSP
Time (min)
P
NMDA/AMPA (%)

**Fig. S4. The surface accumulation of GluA1-homomers upon CALM loss is due to an endocytic defect.**

(**A**) Illustration of breeding scheme used for the generation of tamoxifen-inducible CALM KO mice (CALM$^{CAG}$). (**B**) Efficient depletion of CALM by inducible genetic knockout (CALM$^{CAG}$) or shRNA-based knockdown (CALM$^{KD}$). Lysates obtained from CALM WT and CALM KO$^{CAG}$ hippocampal neuron cultures or from WT neurons transduced with lentivirus encoding either an inactive scrambled shRNA (CTRL with co-expression of either mKate or GFP to identify transduced cells) or an anti-CALM shRNA (CALM$^{KD}$ with co-expression of either mKate or GFP) were analyzed by immunoblotting with antibodies against CALM and SNAP-25 (as loading control). Representative immunoblot and quantification are shown. CALM protein levels in KO or KD conditions were normalized to WT or scrambled control, respectively. Data represent mean±SEM (n=at least 5 independent experiments; one sample t-test). (**C**) Schematic representation of acid-base quenching experiments using pHluorin-tagged proteins. In neutral buffer surface-localized pHluorin molecules are fluorescent. Applying acidic buffer quenches the pHluorin surface pool. Neutral buffer is used to wash away the acidic buffer prior to unquenching the total pool of pHluorin-tagged molecules with a basic buffer comprising ammonium chloride. (**D** and **E**) The surface pool of GluA1, but not GluA2 is affected by CALM knockdown. Surface/total protein ratio was assessed by acid-base quenching of pHluorin-tagged GluA1 (D) and GluA2 (E) in WT hippocampal neurons expressing scrambled shRNA (CTRL) or an anti-CALM shRNA (CALM$^{KD}$) in combination with mKate (for identification of transduced cells). Data represent mean±SEM (n=4 independent experiments for GluA1 and n=3 independent experiments for GluA2; two-tailed unpaired t-test). (**F**) CALM WT is efficiently expressed via viral (AAV2/9) transduction. Representative immunoblot of lysates from WT and CALM KO$^{CAG}$ hippocampal neurons transduced with pAAV-iRFP (as control) or pAAV-CALM-WT and probed with CALM antibody. Ponceau S staining is shown as loading control. (**G** and **H**) Confirmation of AP180 protein loss in AP180 KO mice. Lysates from hippocampal neuron cultures of WT and AP180 KO mice were immunoblotted with the indicated antibodies. Representative immunoblot (G) and quantification (H) are shown. AP180 levels in KO cultures (which are close to background noise) were normalized to AP180 levels in WT (H) (n=4 independent experiments; one-sample t-test). (**I**) The abundance of GluA1 on the neuronal surface is not altered in absence of AP180. Surface/total protein ratio assessed by acid-base quenching of pHluorin-tagged GluA1 in WT and AP180 KO hippocampal neurons. Data represent mean±SEM (n=6 independent experiments; two-tailed unpaired t-test). (**J** and **K**) Knockout or knockdown of CALM does not affect the surface level of synaptophysin. Surface/total protein ratios assessed by acid-base quenching of synaptophysin-pHluorin expressed in WT and CALM KO$^{CAG}$ hippocampal neurons (J) or WT neurons transduced with a

lentivirus expressing scrambled shRNA (CTRL) or an anti-CALM shRNA ($CALM^{KD}$) in combination with mKate (to identify transduced cells) (K). Data represent mean±SEM (n=5 independent experiments for $CALM^{CAG}$; n=3 independent experiments for $CALM^{KD}$; two-tailed unpaired t-test). (**L** to **N**) Loss of CALM leads to increased surface levels of endogenous GluA1, but not GluA2. Cell surface proteins of WT cortico-hippocampal neuron cultures expressing scrambled shRNA (CTRL) or anti-CALM shRNA ($CALM^{KD}$) were biotinylated and affinity-purified using streptavidin beads. Total and biotinylated proteins (surface) were analyzed by immunoblotting. Quantification of surface protein levels relative to total protein levels for KD neurons is depicted in (L). The surface/total ratio of $CALM^{KD}$ was normalized to CTRL cells. Total protein levels for the biotinylation experiment with knockout cells (depicted in Figure 4D and E) are shown in (M), instead total levels for the experiment with knockdown neurons in (N) (n=at least 3 independent experiments; one sample t-test). (**O** and **P**) Loss of CALM does not affect NMDAR function. NMDAR-mediated fEPSPs were isolated using the AMPA/Kainate receptor antagonist NBQX (10 μM), the $GABA_A$/glycine receptor antagonist picrotoxin (50 μM) and by reducing the $Mg^{2+}$ concentration to 0.25 mM. Initial AMPA responses were taken as 100%. Isolated NMDA responses measured between 40-50 min after application of antagonists were calculated and compared to initial AMPA responses. NMDA receptor antagonist APV (50 μM) was applied to verify NMDA responses. Representative WT and CALM $KO^{EMX}$ fEPSPs of AMPA (solid line) and NMDA responses (dashed line) as well as remaining traces after application of the NMDA antagonist APV (dotted line) are shown above (scale bars: 1 mV and 10 ms) (O). The bar diagrams of NMDA/AMPA ratios in WT and CALM $KO^{EMX}$ show no difference between genotypes (WT N=5 animals, n=12 slices; CALM $KO^{EMX}$ N=5 animals, n=12 slices; two-tailed unpaired t-test) (P).

## Figure S5

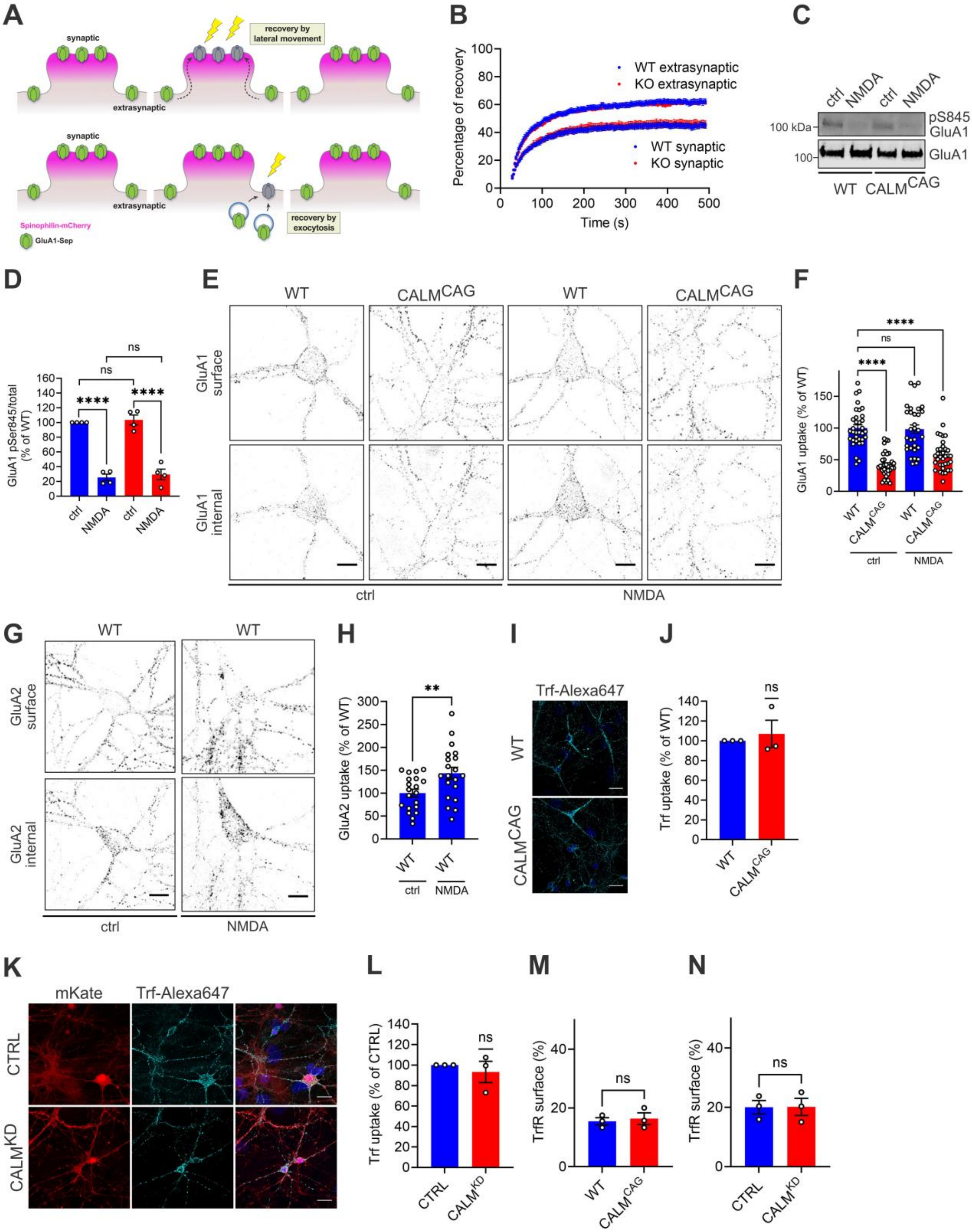
A
synaptic
extrasynaptic
recovery by lateral movement
recovery by exocytosis
Spinophilin-mCherry
GluA1-Sep
B
Percentage of recovery
Time (s)
WT extrasynaptic
KO extrasynaptic
WT synaptic
KO synaptic
C
ctrl
NMDA
pS845 GluA1
GluA1
100 kDa
WT
CALMCAG
D
GluA1 pSer845/total (% of WT)
ns
****
E
WT
CALMCAG
GluA1 surface
GluA1 internal
ctrl
NMDA
F
GluA1 uptake (% of WT)
****
ns
G
GluA2 surface
GluA2 internal
H
GluA2 uptake (% of WT)
**
I
Trf-Alexa647
J
Trf uptake (% of WT)
K
mKate
CTRL
CALMKD
L
Trf uptake (% of CTRL)
M
TrfR surface (%)
N

**Fig. S5. Loss of CALM does not affect AMPAR lateral diffusion, GluA1 phosphorylation and clathrin-mediated endocytosis of Transferrin receptor.**

(**A**) Experimental design for comparing the effect of synaptic and perisynaptic photobleaching to measure GluA1 mobility. Fully photobleached synapses (top, mCherry-Spinophilin labeled) will only recover fluorescent receptor via lateral diffusion of extrasynaptic receptors. Instead, photobleached extrasynaptic regions (bottom) will recover fluorescent receptor via exocytosis of GluA1-containing endosomes. (**B**) The surface accumulation of GluA1 upon CALM loss is not due to increased exocytosis or alterations in lateral diffusion. WT and CALM KO$^{CAG}$ hippocampal neurons co-expressing pHluorin-tagged GluA1 and the dendritic marker mCherry-Spinophilin were photobleached. Fluorescence recovery after photobleaching (FRAP) was quantified as readout for exocytic delivery or lateral diffusion of unbleached pHluorin-GluA1 molecules. Exemplary recovery curves following synaptic (bottom curves) or extrasynaptic (top curves) photobleaching. Quantification in Fig. 4I. (**C** and **D**) LTD-induced GluA1-pS845 dephosphorylation is unaltered upon CALM loss. Lysates of WT and CALM KO$^{CAG}$ hippocampal neurons subjected to chemical LTD induction were analyzed by immunoblotting with the indicated antibodies (C). GluA1-pS845 was normalized for the total levels of GluA1 (D) (n=4 independent experiments; one-way ANOVA followed by Dunnett´s post hoc test). (**E-H**) GluA1 endocytosis in absence of CALM remains impaired upon NMDA application. WT and CALM KO$^{CAG}$ hippocampal neurons overexpressing SEP-GluA1 (E) or WT neurons overexpressing SEP-GluA2 (G) were live-labelled using anti-GFP antibodies. After 15 min at 37°C to allow internalization in the absence or presence of NMDA, neurons were fixed. Immunostaining was performed either in absence or presence of detergent to label the surface or the internal GluA1/A2 pools. Representative immunofluorescences (scale bar, 10 µm) (E and G) and quantification (F and H) are shown. Data represent mean±SEM (each data point represents a neuron from n=4 independent experiments for SEP-GluA1 and n=3 independent experiments for SEP-GluA2, two-tailed unpaired t-test). (**I-L**) Knockout or knockdown of CALM does not affect Transferrin uptake. WT and CALM KO$^{CAG}$ hippocampal neurons (I and J) or WT neurons transduced either with scrambled shRNA (CTRL) or anti-CALM shRNA (CALM$^{KD}$) in combination with mKate (to identify transduced cells) (K and L) were incubated with Alexa647-labeled Transferrin for 20 min at 37°C prior to fixation. Representative immunofluorescences (scale bar, 20 µm) (I and K) and quantification (J and L) are shown. Transferrin uptake in CALM$^{CAG}$ or CALM$^{KD}$ neurons was normalized to WT or CTRL cells, respectively. Data represent mean±SEM (n=3 independent experiments, one sample t-test). (**M** and **N**) The abundance of Transferrin receptor (TrfR) on the neuronal surface is not altered upon CALM KO or KD. Surface/total protein ratios of pHuji-tagged TrfR in WT and CALM KO$^{CAG}$ hippocampal neurons (M) or WT neurons transduced with lentivirus encoding scrambled shRNA (CTRL) or

an anti-CALM shRNA ($CALM^{KD}$) in combination with GFP (for identification of transduced cells) (N). Data represent mean±SEM (n=3 independent experiments; two-tailed unpaired t-test).

# Figure S6

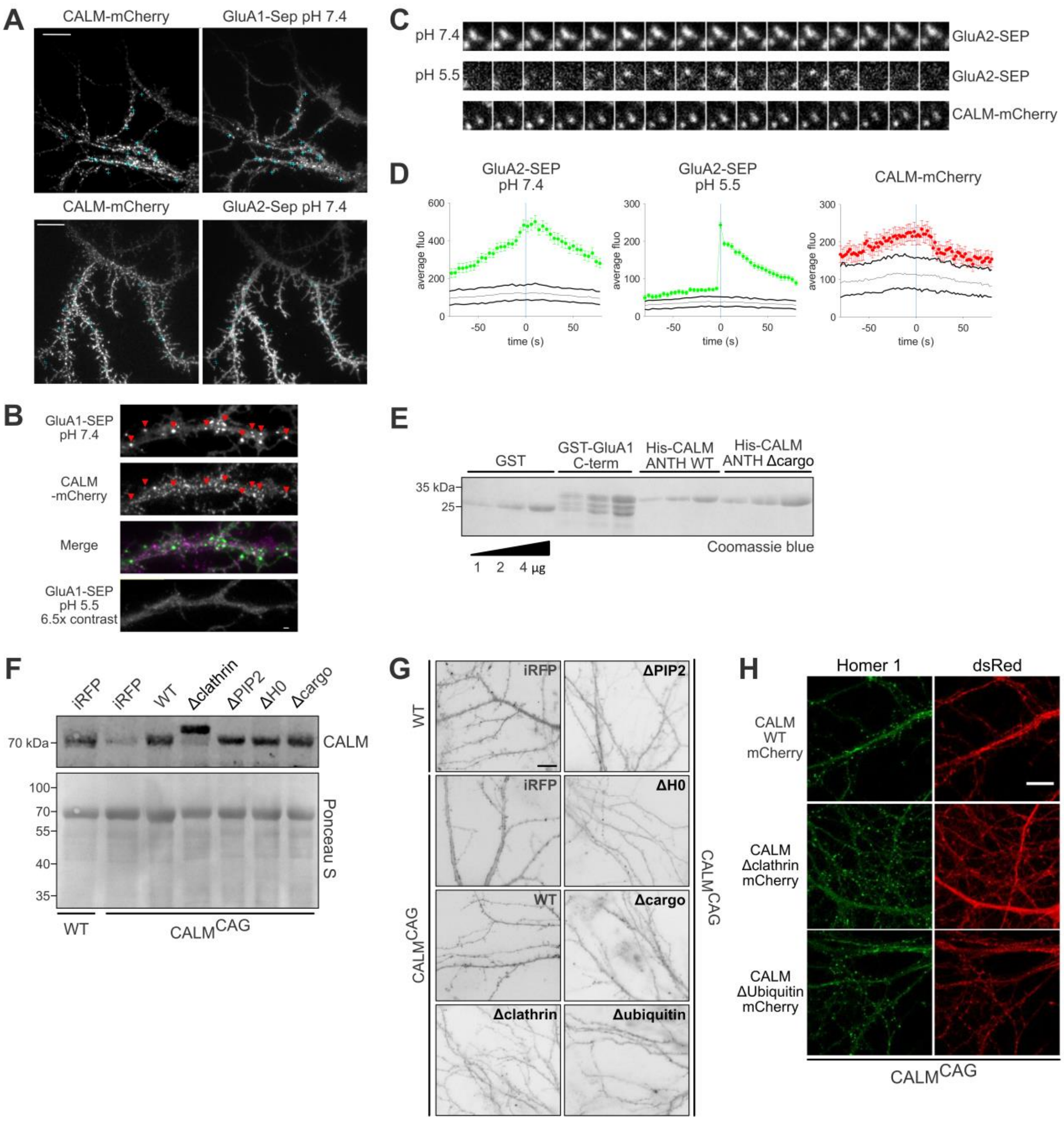


**Fig. S6. CALM is also present at sites of GluA2 endocytosis even though it selectively facilitates the internalization of GluA1.**

(**A**) Map depicting analyzed endocytic events of superecliptic pHluorin (SEP)-GluA1 and SEP-GluA2. Events were detected in dendrites of a neuron transfected with SEP-GluA1 or SEP-GluA2 and CALM-mCherry and imaged for 5 min. The sites of events detected during the

recording time are overlaid on the corresponding CALM-mCherry and SEP-GluA1 or SEP-GluA2 image. (**B**) Co-localization of GluA1 and CALM. Depiction of a portion of dendrite of a rat neuron (15 DIV) transfected with SEP-GluA1 and CALM-mCherry. Top, SEP-GluA1 at pH 7.4. Most clusters corresponding to PSDs (red arrowheads) are next to CALM-mCherry clusters. Other CALM clusters are located further away from PSDs. Bottom, SEP-GluA1 at pH 5.5 at 6.5x contrast compared to the image shown at pH 7.4. Colocalization between GluA1-Sep and CALM-mCherry in 15 neurons was evaluated by Pearson's R coefficient: 0.55 ± 0.04 (which is above the randomized controls: 0.23 ± 0.02). Scale bar, 2 µm. (**C** and **D**) CALM is also present at sites of GluA2 endocytosis. (C) Example of a GluA2 endocytic event detected with the ppH assay. Images taken at pH 7.4 (top) and 5.5 (middle) at times relative to time 0, the moment of vesicle detection at pH 5.5. (D) Average fluorescence intensity (fluo.) of 324 endocytic events detected in 15 cells in the green and red channels. The fluorescence intensity is aligned to the time of vesicle detection (frame 0). Gray areas represent the 95% confidence interval of randomized data indicating a clear CALM enrichment at the site of vesicle formation. (**E**) Efficient expression and purification of GST, GST-tagged GluA1 C-terminal tail and His-tagged WT and cargo-binding deficient (Δcargo) CALM ANTH domain. 1, 2, or 4 µg of purified protein were separated by SDS-PAGE and stained with Coomassie blue. (**F**) Comparable expression levels of mutant CALM variants. Lysates of WT and CALM KO$^{CAG}$ hippocampal neurons transduced with adeno associated virus AAV2/9 to express either iRFP (as control), CALM-WT or mutant versions of CALM (Δclathrin, clathrin-binding deficient; ΔPIP2, $PI(4,5)P_2$-binding deficient; ΔH0, membrane insertion deficient; Δcargo, cargo binding deficient) were probed by immunoblotting to verify the expression of the rescue constructs. Note that CALM mutated in 8 amino acids to abrogate clathrin binding shows altered running behavior. Ponceau S staining is shown as loading control. (**G**) Overall neuronal growth and health as well as spine morphology are unaffected by viral CALM expression. Hippocampal neurons were transduced at DIV2 with adeno-associated virus (AAV2/9) to express either iRFP (as control), CALM-WT or mutant versions of CALM. At DIV7 neurons were transfected with SEP-GluA1. Representative images taken at DIV14 of SEP-GluA1 at pH 7.4 are shown. (**H**) CALM localization is unaffected upon mutation of clathrin and ubiquitin binding sites. CALM KO$^{CAG}$ hippocampal neurons transduced with AAV2/9 to express either CALM-WT, CALM Δclathrin or Δubiquitin were stained with the indicated primary antibodies at DIV14 (scale bar: 20 µm).

**Figure S7**

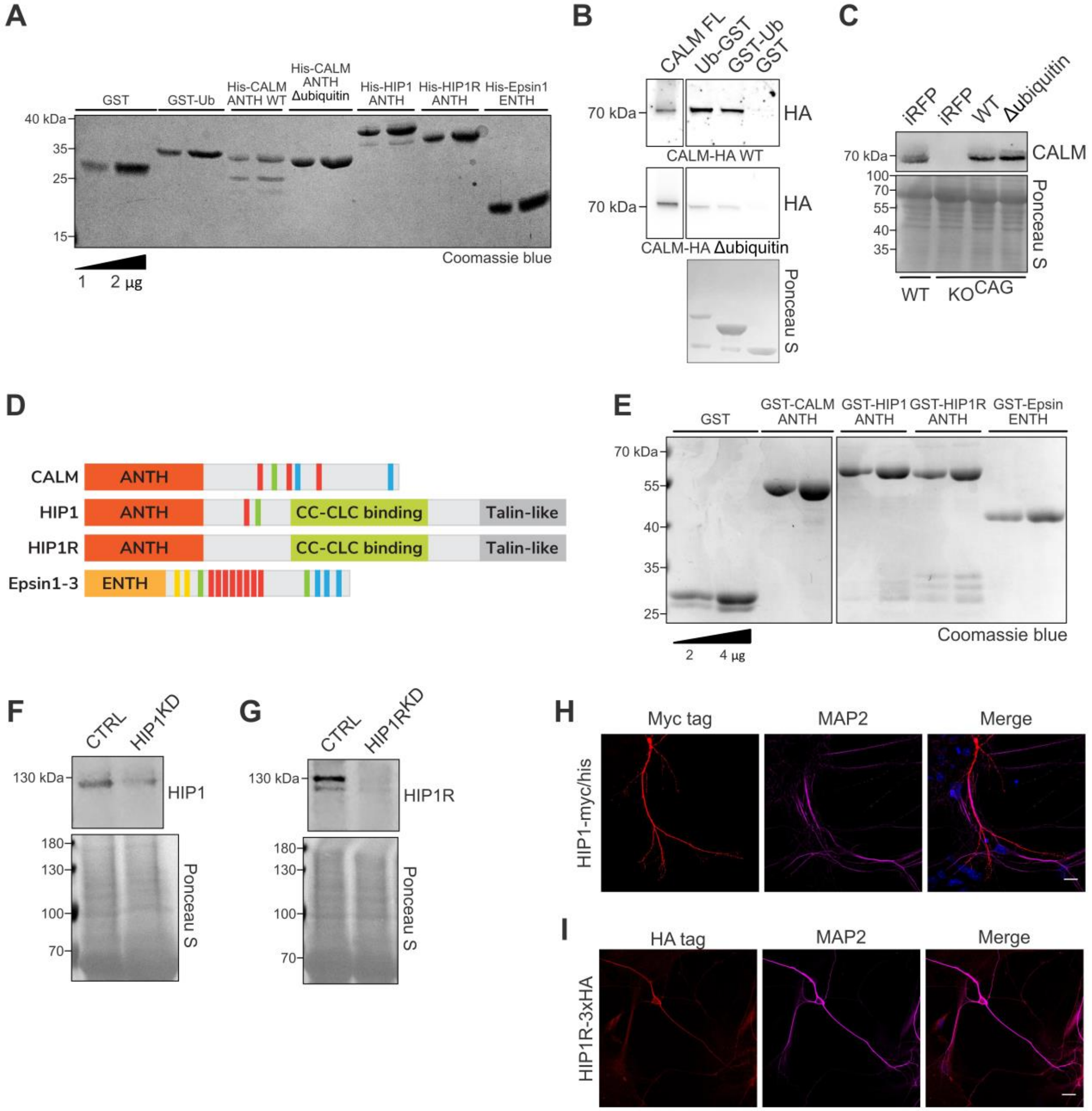


**Fig. S7. Role of ANTH domain proteins and ubiquitination in the endocytosis of GluA1.**

(**A**) Efficient expression and purification of GST, GST-ubiquitin and His-tagged ANTH domains of CALM WT, CALM ubiquitin-binding deficient (Δubiquitin), HIP1, HIP1R and His-tagged ENTH domain of Epsin1. 1 or 2 µg of purified protein were separated by SDS-PAGE and stained with Coomassie blue. (**B**) Full length CALM binds to ubiquitin. N-terminal or C-terminal GST-tagged ubiquitin or GST alone were incubated with purified HA-tagged full-length CALM

or an ubiquitin-binding deficient mutant (Δubiquitin). After extensive washing, GST-tagged proteins and CALM ANTH domain retention were analyzed by immunoblotting. (**C**) Comparable expression levels of WT and ubiquitin-binding deficient CALM. Lysates of WT and CALM $KO^{CAG}$ hippocampal neurons transduced with AAV2/9 to express either iRFP (as control), CALM-WT or a ubiquitin-binding deficient mutant of CALM (Δubiquitin) were probed by immunoblotting to verify the expression of the rescue constructs. (**D**) Overview of domain structure of ANTH and ENTH domain containing proteins. (**E**) Efficient expression and purification of GST, GST-tagged ANTH and ENTH domains. 2 or 4 µg of purified protein were separated by SDS-PAGE and stained with Coomassie blue. (**F** and **G**) Efficient depletion of HIP1 (F) and HIP1R (G) by shRNA. Lysates of WT hippocampal neurons expressing scrambled shRNA (CTRL) or anti-HIP1 shRNA ($HIP1^{KD}$) or anti-HIP1R shRNA ($HIP1R^{KD}$) were probed by immunoblotting for the indicated proteins. Ponceau S staining is shown as loading control. (**H** and **I**) Efficient over-expression of HIP1 and HIP1R. WT hippocampal neurons transfected with HIP1-Myc/His (H) or HIP1R-3xHA (I) encoding constructs were stained with the indicated primary antibodies (scale bar: 20 µm).
